# An ionic vinylene-linked three-dimensional covalent organic framework for selective and efficient trapping of ReO_4_^−^ or ^99^TcO_4_^−^

**DOI:** 10.1038/s41467-022-35435-7

**Published:** 2022-12-09

**Authors:** Cheng-Rong Zhang, Wei-Rong Cui, Shun-Mo Yi, Cheng-Peng Niu, Ru-Ping Liang, Jia-Xin Qi, Xiao-Juan Chen, Wei Jiang, Xin Liu, Qiu-Xia Luo, Jian-Ding Qiu

**Affiliations:** 1 College of Chemistry and Chemical Engineering, Nanchang University, 330031 Nanchang, China; 2 State Key Laboratory of Nuclear Resources and Environment, East China University of Technology, 330013 Nanchang, China

**Keywords:** Organic molecules in materials science, Polymers, Synthesis and processing

## Abstract

The synthesis of ionic olefin linked three-dimensional covalent organic frameworks (3D COFs) is greatly challenging given the hardness of the formation of stable carbon-carbon double bonds (–C = C–). Herein, we report a general strategy for designing porous positively charged sp^2^ carbon-linked 3D COFs through the Aldol condensation promoted by quaternization. The obtained 3D COFs, namely TFPM-PZI and TAPM-PZI, showed impressive chemical stability. Furthermore, the positively charged frameworks with regular porosity endow 3D ionic COFs with selective capture radioactive ReO_4_^−^/TcO_4_^−^ and great removal efficiency in simulated Hanford waste. This research not only broadens the category of 3D COFs but also promotes the application of COFs as efficient functional materials.

## Abstract (web summary)

The synthesis of ionic olefin linked threedimensional covalent organic frameworks (3D COFs) is challenging because of the difficulties in forming stable carbon-carbon double bonds. Here, the authors report a general synthesis of an ionic sp^2^ carbon linked COFs and demonstrate selective capture of ReO_4_^−^ ions.

## Introduction

Covalent organic frameworks (COFs) represent a type of porous crystalline materials linked by strong covalent bonds between light atoms^1^. Unlike traditional polymers^1^, COFs can accurately organize molecular species into two- or three-dimensional (2D or 3D) networks with structural periodicity and inherent porosity^2–5^. To date, most research has concentrated on 2D architectures with eclipsed AA or staggered AB stacking patterns^6,7^, and reports of 3D COFs are greatly restricted. Generally, 2D COFs only have uniform one-dimensional channels, while 3D COFs have more complex pore structures with more void frameworks, which are more conducive to separation^8^, catalysis^9^, guest binding^10^, etc. Furthermore, high surface area, low density, and abundant easily available active sites are often observed in 3D COFs due to more voided frameworks, which is hardly achievable for 2D COFs^2,3,11^. Unlike 2D COFs, topology is a crucial parameter that determines the pore structure, properties and potential applications of 3D COFs^12^. However, so far, there have been only single-digit different topologies in 3D COFs (ctn, bor, dia, pts, rra, srs, ffc, and lon) and the exploration of additional structures is still the frontier in this field^13,14^. Among all 3D COFs synthesis strategies, the most commonly used dynamic covalent bond relies on imine bond and boronate ester bond^15,16^, exhibiting relatively poor stability and weak electron delocalization, which severely hinder their practical applications^17^. Therefore, the identification of structures and the development of connection covalent bond are still major challenges of 3D COFs.

The emergence of olefin-linked COFs overcomes the current limitations of dynamic covalent bond formation^17–19^. The irreversible of C = C bond enables the framework to exhibit impressive stability under harsh conditions, and offers extended π conjugation throughout the framework for efficient electron transport, showing great potential applications in photocatalysis^20–23^. So far, the only example of 3D sp^2^ carbon-linked COFs involves substituted acrylonitrile [−CH = C(CN)−] based on the Knoevenagel condensation between arylacetonitriles and aromatic aldehydes^17,24^. Nevertheless, the strong electron-withdrawing substitution of nitriles attached to the olefin bond makes the bond reversible, compromising the stability and the crystallinity of COFs^25^. It has been reported that unsubstituted vinylidene bonds (−CH = CH−) tend to form well-defined in-plane structures, thereby improving the crystallinity and physicochemical properties of materials^26,27^. This provides a strategy for synthesizing more stable 3D sp^2^ carbon-linked COFs and broadening the types of 3D COFs. Moreover, almost all 3D COFs exhibit neutral backbones due to a the limitations of building blocks and complex post-modifications processes^3,11^, hence the synthesis of ion-functionalized 3D COFs is also considered as a great challenge^1,28,29^.

^99^Tc, technetium is a long-lived (*t*_1/2_ = 2.13 × 10^5 ^y) radioactive isotope, a rich nuclear waste component, and a source of strong radioactive pollution^30–33^. Non-radioactive ReO_4_^−^ is often regarded as a surrogate for the chemical behavior of TcO_4_^−^, since they have similar thermodynamic parameters, electronic distribution, and spatial configuration^32,33^. So far, ion exchange has become one of the most promising methods for capturing ReO_4_^−^/TcO_4_^−^, due to its simple process, environmental friendliness and low cost^34–36^. Many cationic materials have been used to capture ReO_4_^−^/TcO_4_^−^^37,38^, while the slow adsorption kinetics and poor chemical stability are still inevitable problems. As well as in the case of a large amount of SO_4_^2−^ and NO_3_^−^ in the natural waste system, the selective capture of ReO_4_^−^/TcO_4_^−^ is a major scientific and technical challenge^39–41^.

Taking these considerations in mind, we herein report a general strategy for constructing ionic 3D sp^2^ carbon-linked COFs (TAPM-PZI and TFPM-PZI) through acid-catalyzed Aldol condensation between tetra(4-formylphenyl)methane (TFPM), 1,3,5,7-tetrakis(4-aldophenyl)-adamantane (TAPM) and 1,2,5-trimethylpyrazin-1-ium iodide (PZI), respectively. The synthesized COFs exhibited impressive chemical stability. In addition, the unique 3D building units with high symmetry uniformly separate positive charges, making the synthesized COF as an ideal platform for the rapid removal of nuclear waste ions (ReO_4_^−^/TcO_4_^−^). We highlight that this sp^2^ carbon-linked 3D COF will open a window for the design and synthesis of porous crystalline functional materials.

## Results

### Fabrication and characterization of COFs

**Fig. 1:**
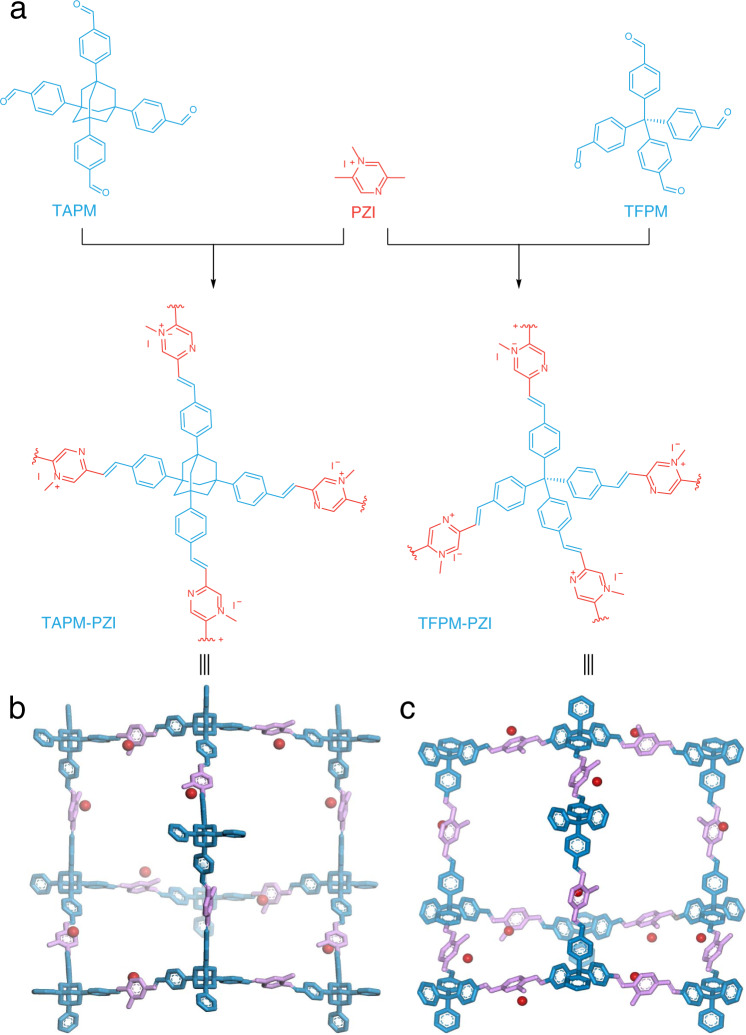
Schematic of synthetic ionic 3D sp^2^ carbon-linked COFs. **a** Schematic synthesis of the ionic vinylidene-linked 3D COFs. The adamantane-knotted cage in the diamond net of **b** TAPM-PZI and **c** TFPM-PZI, respectively.

The synthesis of 3D sp^2^ carbon-linked COFs was based on the Aldol condensation promoted by quaternization^27^. TAPM and TFPM were designed as a tetrahedral building unit (Fig. 1a), and PZI was chosen as a linear cationic building block to generate extended 3D framework structures of TAPM-PZI (Fig. 1b) and TFPM-PZI (Fig. 1c), respectively. The highly crystalline TAPM-PZI and TFPM-PZI were obtained in a mixture of mesitylene containing 1,4-dioxane with trifluoroacetic acid as a catalyst, reacted at 150 °C for 72 h (Supplementary Tables 1 and 2 and Supplementary Figs. 1 and 2). Reference model compound A (named Model A) was also synthesized with the same condition. In the FT-IR spectra of 3D sp^2^ carbon-linked COFs (Supplementary Figs. 3 and 4), the vibration band of C = O (ca. 1702 cm^−1^) disappeared. Meanwhile, compared with FT-IR spectra of Model A (Supplementary Fig. 5), the typical C = C vibration band (ca. 1594 cm^−1^) was observed, indicating TAPM-PZI and TFPM-PZI were highly condensed^25^. The ^13^C CP-MAS NMR of COFs further proved highly efficient condensation supported by the peak observed at ca. 134.0 ppm (Supplementary Fig. 6), which was attributed to the formed alkene carbons^21^. Similar spectral changes were also observed for Model A. These results provided strong evidence for the successful formation of carbon–carbon double bond in the 3D framework^42^.

**Fig. 2:**
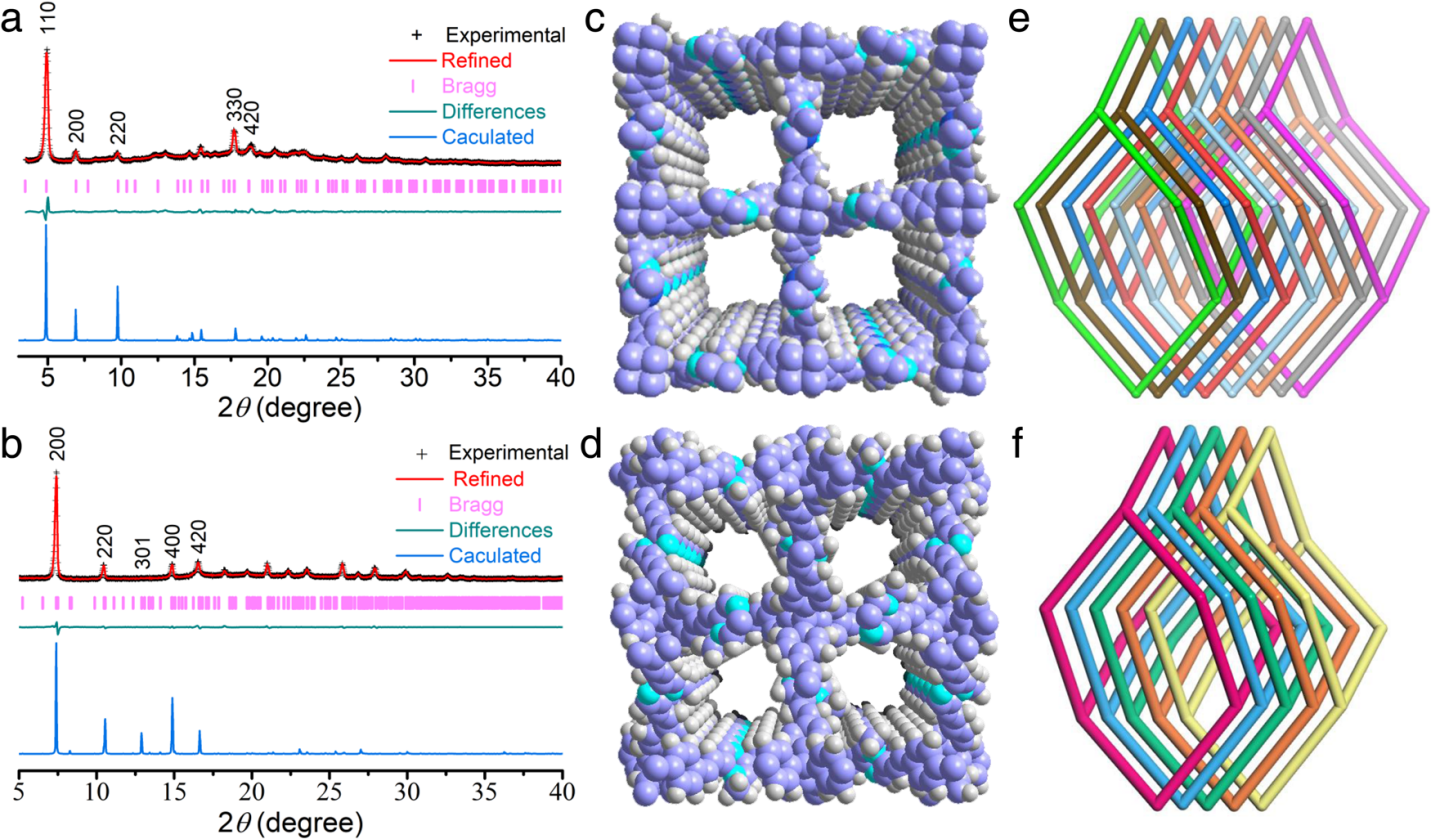
Structural representations of ionic 3D sp^2^ carbon-linked COFs. PXRD patterns of (**a**) TAPM-PZI and (**b**) TFPM-PZI. Structural representations of (**c**) and (**e**) TAPM-PZI, and (**d**) and (**f**) TFPM-PZI, respectively (C, purple; H, grey; N, sapphire; I, navy blue).

The crystallinities of ionic 3D sp^2^ carbon-linked COFs were evaluated by powder X-ray diffraction (PXRD) combined with structure simulation (Fig. 2a, b). After a geometrical energy minimization by the Materials Studio software package based on eightfold and fivefold interpenetrated dia net for TAPM-PZI and TFPM-PZI, respectively, their unit cell parameters were obtained (*a* = *b* = 25.5472 Å, *c* = 6.3857 Å and *α* = *β* = *γ* = 90° for TAPM-PZI; *a* = *b* = 28.8894 Å, *c* = 13.5320 Å and *α* = *β* = *γ* = 90° for TFPM-PZI; Supplementary Tables 3 and 4). The simulated PXRD patterns were matched well with the experimental ones^3^. Peaks at 4.95°, 6.95°, 9.80°, 14.67°, and 15.43° for TAPM-PZI belong to the (110), (200), (220), (330), and (420) Bragg peaks of space group *P*−4 (No. 81), respectively; peaks at 7.41°, 10.48°, 12.88°, 14.84°, and 16.58° for TFPM-PZI were assigned to the (200), (220), (301), (400), and (420) Bragg peaks of the space group *P*−4 (No. 81), respectively. Moreover, full profile pattern matching (Pawley) refinements were performed for both ionic 3D COFs. The refinement results matched well with the experimental results with a negligible difference and good agreement factors (*Rp* ≤ 0.95% and *Rwp* ≤ 1.98% for TAPM-PZI; *Rp* ≤ 0.23% and *Rwp* ≤ 0.64% for TFPM-PZI). Based on the above results, the synthesized COFs were proposed to have the anticipated architectures with eightfold, fivefold interpenetrated dia net, showing microporous cavities with a diameter of about 14.5 Å for TAPM-PZI, 7.8 Å for TFPM-PZI, respectively (Fig. 2c–f).

**Fig. 3:**
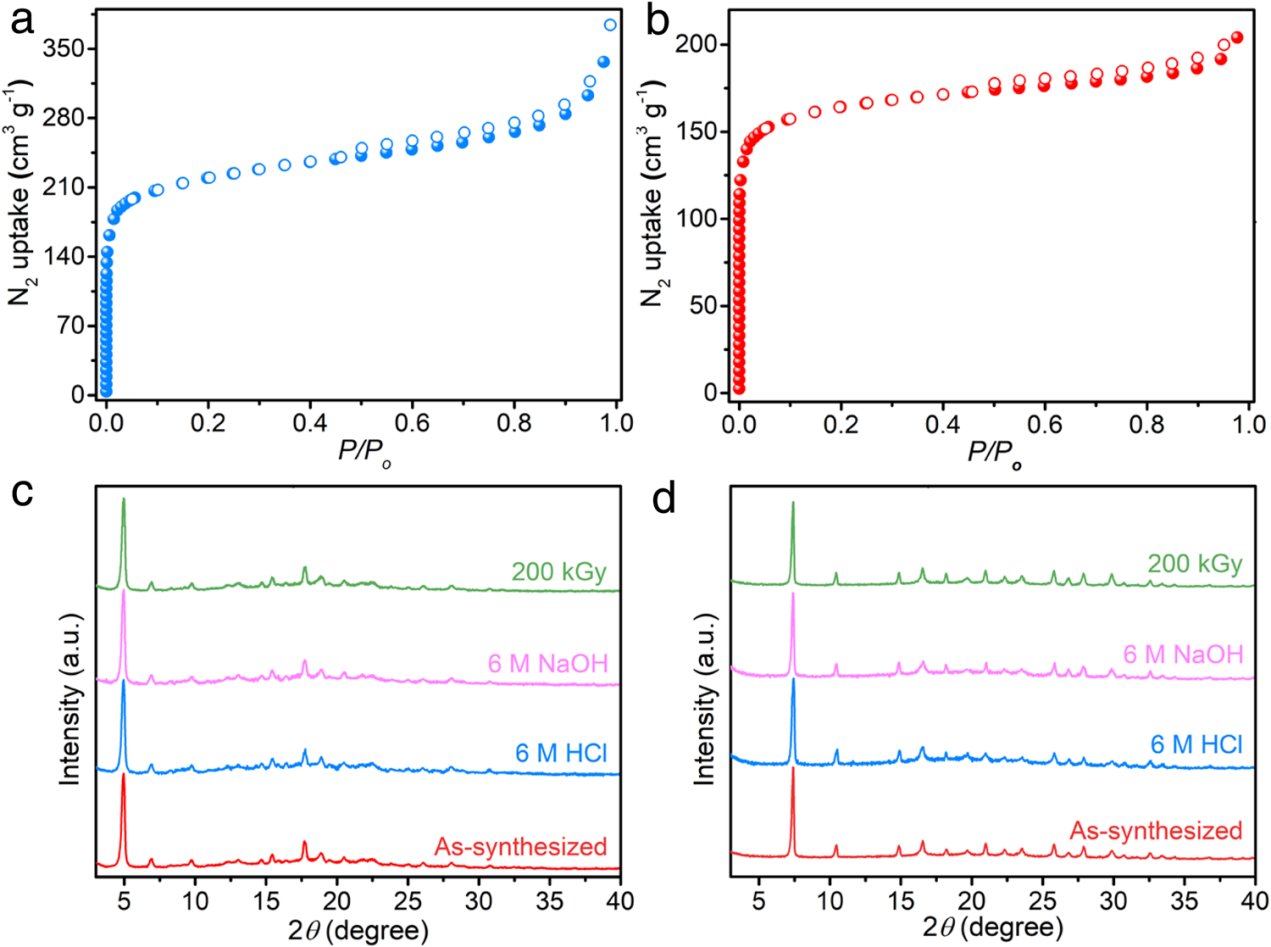
Characterization and stability of 3D sp^2^ carbon-linked COFs. N_2_ adsorption-desorption isotherms for (**a**) TAPM-PZI and (**b**) TFPM-PZI. PXRD patterns of (**c**) TAPM-PZI and (**d**) TFPM-PZI before and after treatment with 200 kGy γ-ray irradiation, 6 M NaOH, 6 M HCl for 48 h.

The permanent porosities of ionic 3D sp^2^ carbon-linked COFs were investigated by nitrogen adsorption−desorption isotherm at 77 K. Both COFs showed a classic type I isotherm, indicating its microporous feature. The Brunauer–Emmett–Teller (BET) surface areas of TAPM-PZI and TFPM-PZI were analyzed to be 598.3 m^2^ g^−1^ and 501.6 m^2^ g^−1^, respectively (Fig. 3a, b and Supplementary Figs. 7 and 8). The relatively lower surface area of TFPM-PZI and TAPM-PZI could be attributed to increased amorphous material with relatively low porosities and the presence of counter ions, where part of the material’s pores is occupied by counter anions, further leading to pore blockage^43^. According to the nonlocal density function theory (NLDFT) method, the pore size distribution was centered at 1.21 nm and 0.71 nm for TAPM-PZI and TFPM-PZI, respectively (Supplementary Figs. 9 and 10), which were consistent with the pore size forecasted from crystal structures. The ionic properties of as-synthesized COFs were characterized by Zeta potential analysis. Both COFs exhibited positive values over a wide pH range (2–10), demonstrating their positively charged skeletons (Supplementary Fig. 11). Furthermore, the scanning electron microscope (SEM) and transmission electron microscopy (TEM) images revealed a uniform spherical morphology of TAPM-PZI and TFPM-PZI (Supplementary Figs. 12–15). High-resolution transmission electron microscope (HRTEM) images of TAPM-PZI and TFPM-PZI showed clear lattice fringes, confirming their long-range order structures^44^ (Supplementary Figs. 16 and 17).

Furthermore, it was worth noting that the TAPM-PZI and TFPM-PZI also exhibited great chemical stability with 6 M NaOH, 6 M HCl and strong radiation (Fig. 3c, d and Supplementary Figs. 18 and 19). Thermogravimetric analysis (TGA) curves exhibited no significant weight loss even at temperatures up to 450 °C (Supplementary Figs. 20 and 21), indicating the high thermal stability of TAPM-PZI and TFPM-PZI. The great stability of as-synthesized COFs could be attributed to the existence of carbon-carbon double bonds, which can effectively protect the hydrolytically susceptible framework and provide a prospect for the practical application of the COF.

We further characterized the optical properties of TAPM-PZI and TFPM-PZI. Compared with the Model A and monomers (TAPM and TFPM), as-synthesized COFs process a broader absorption spectrum in the ultraviolet-visible diffuse reflectance spectroscopy (UV-vis DRS) with an absorption edge of 1200 nm (Supplementary Figs. 22 and 23). In addition, the average lifetimes of TAPM-PZI and TFPM-PZI and Model A were analyzed by the time-resolved fluorescence decay curves to be 1.66, 1.86, and 0.42 ns, respectively (Supplementary Fig. 24). Compared with monomers and Model A, the favorable optical properties of as-synthesized COFs may be due to the formation of vinylidene bonds that provide a π-conjugated scaffold, thereby improving the π-electron delocalization in the 3D frameworks^21,45–47^. In addition, the electronic properties of as-synthesized COFs and Model A were further studied by a series of electrochemical experiments. Electrochemical impedance spectroscopy (EIS) of ionic sp^2^ carbon-linked 3D COFs showed smaller impedances and higher low-frequency slopes than those of Model A and the reported 2D sp^2^ carbon-linked COFs (SP^2^c-COF)^19^ (Supplementary Figs. 25 and 26), indicating that the interfacial charge transport resistance was reduced upon the formation of ionic sp^2^ carbon-linked 3D COFs, which will be beneficial for the carriers to be rapidly transported. The much higher transient photocurrent response of TAPM-PZI and TFPM-PZI than that of Model A further supported this conclusion (Supplementary Fig. 27). In addition, both COFs showed ideal capacitance behavior^48,49^ (Supplementary Figs. 28 and 29). We highlight that the synthesized sp^2^ carbon-linked 3D COF will open a window for the design and synthesis of porous crystalline materials with photocatalytic properties.

### ReO_4_^−^ adsorption performance of TFPM-PZI

**Fig. 4:**
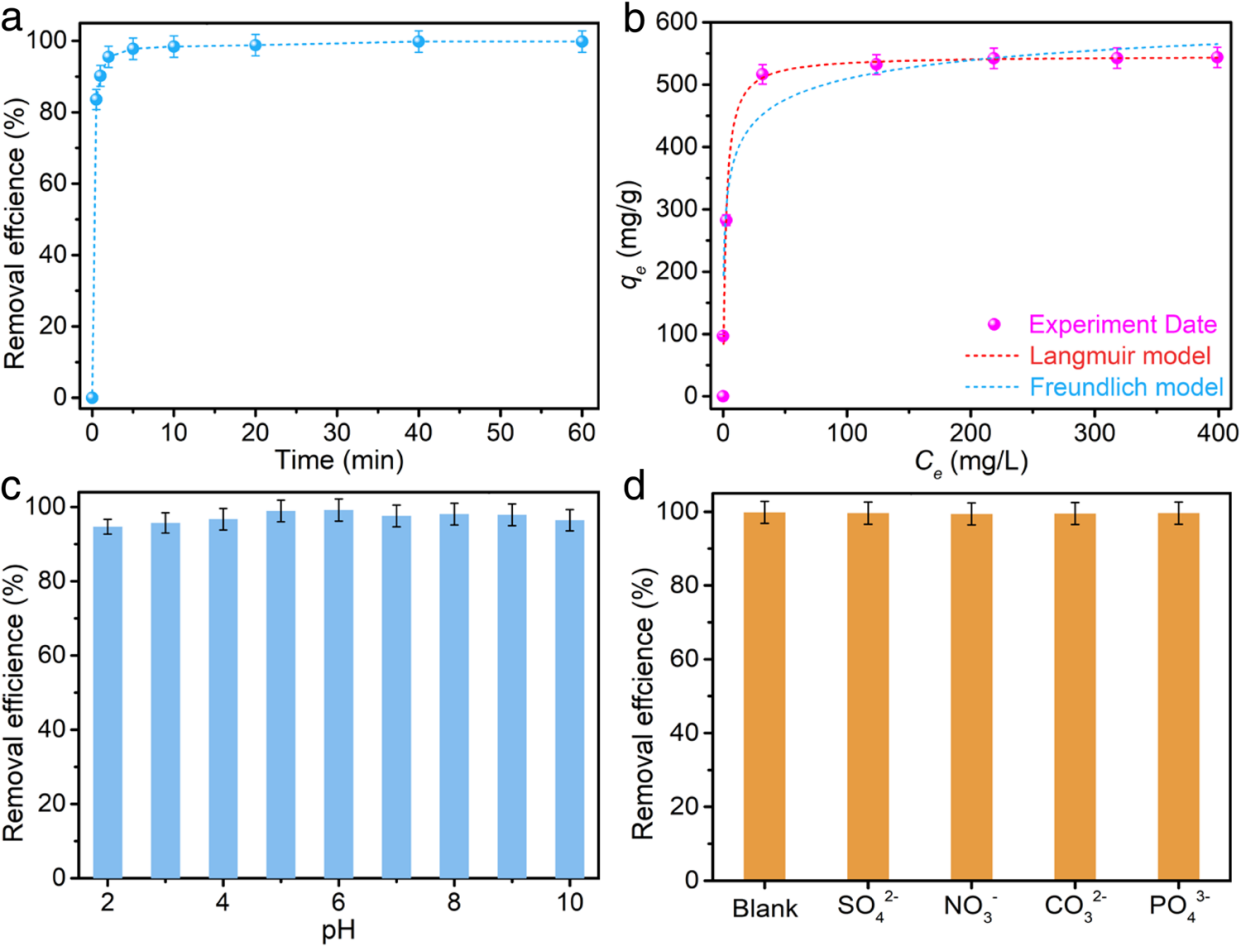
ReO_4_^−^ adsorption isotherms and kinetics investigations. **a** Adsorption kinetics of ReO_4_^−^ by TFPM-PZ-Cl. **b** Adsorption isotherm of TFPM-PZ-Cl for ReO_4_^−^ capture. **c** Effect of pH on ReO_4_^−^ capture by TFPM-PZ-Cl. **d** Removal efficiency of ReO_4_^−^ by TFPM-PZ-Cl in the presence of competitive anions. Error bars represent S.D. *n* = 3 independent experiments.

In view of the high crystallinity, regular porosity, impressive chemical stability, and ionic nature of as-synthesized 3D COFs. Here in, we took the TFPM-PZI as an example to investigate its potential for capturing radioactive technetium (Tc-99) in nuclear waste. ReO_4_^−^ was used as a non-radioactive substitute to simulate radioactive TcO_4_^−^. TFPM-PZI was immersed in saturated NaCl solution overnight to remove toxic I^−^ to obtain TFPM-PZ-Cl (Supplementary Figs. 30 and 31)^40,50^. Batch adsorption experiments showed when the ReO_4_^−^ concentration was 28 ppm, TFPM-PZ-Cl could almost quantitatively remove ReO_4_^−^ from the solution within 30 s (Fig. 4a), which was better than those of most reported adsorbents (Supplementary Table 5). Meanwhile, the adsorption capacity of ReO_4_^−^ reached 542.3 mg g^−1^ (Fig. 4b and Supplementary Table 6)^51^. In addition, even in an aqueous solution of pH 2, TFPM-PZ-Cl still had a removal efficiency of >90% (Fig. 4c), which may be attributed to the positive charges on the surface of TFPM-PZI over a wide pH range (2–10), which can efficiently trap anions (Supplementary Fig. 11). Compared with all existing anion exchange materials^52,53^, TFPM-PZ-Cl significantly enhanced acid and alkali resistance. This powerful advantage stems from the impressive stability of the material given by the olefin bond.

Despite the exist of a high concentration of competing anions, TFPM-PZ-Cl still had great selectivity to ReO_4_^−^ (Fig. 4d and Supplementary Figs. 32 and 33). In addition, even after five cycles, TFPM-PZ-Cl still remained nearly 97.6% removal efficiency at the initial concentration of 28 ppm (Supplementary Fig. 34), and the recovered SEM image and PXRD pattern further revealed the high stability of the material, making it very cost-effective in separation applications (Supplementary Figs. 35 and 36). Meanwhile, in the simulated Hanford LAW scrubber solution (Supplementary Table 7), when the adsorbent/solution ratio was 5 mg mL^–1^, the removal efficiency of ReO_4_^−^ could reach 86.4% (Supplementary Table 8), which is notably better than those of the recently reported bis-PC6@TbDp-COF (77%)^51^, SCU-CPN-1-Br (83.7%)^54^, and SCU-COF-1 (56.3%)^55^. Surprisingly, the concentration of ReO_4_^−^ after column adsorption was about 10 ppb, and the removal rate of ReO_4_^−^ was as high as 99.99% (Supplementary Fig. 37). Therefore, TFPM-PZ-Cl is an ideal material for removing ReO_4_^−^/TcO_4_^−^ from industrial wastewater.

### Adsorption mechanism of ReO_4_^−^

**Fig. 5:**
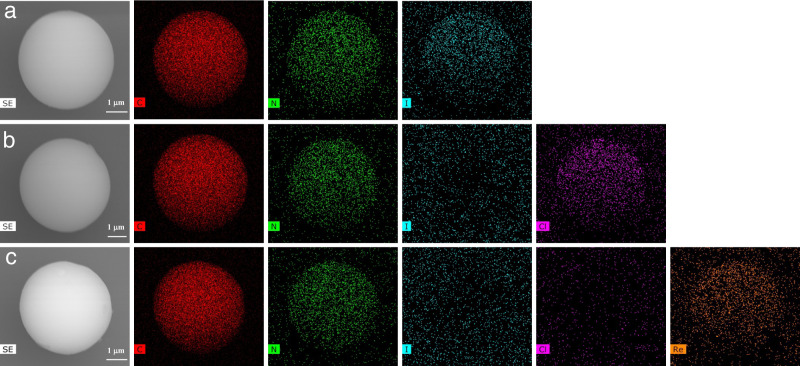
EDS mapping. **a** TFPM-PZI, **b** TFPM-PZ-Cl, **c** TFPM-PZ-Re. C (red), N (green), I (blue), Cl (pink), Re (yellow).

The anion exchange process of TFPM-PZ-Cl was further investigated using FT-IR, Raman spectroscopy, X-ray photoelectron spectroscopy (XPS) and energy dispersive X-ray spectroscopy (EDS) mapping analysis. After ReO_4_^-^ adsorbed, a peak at 909 cm^−1^ (Re–O bond) appeared in the FT-IR spectrum (Supplementary Fig. 38)^52^. In addition, from the Raman spectra, the peaks at 956 cm^−1^ and 330 cm^−1^ for ReO_4_^−^ (NaReO_4_ as reference), indicated the Re was successfully captured by TFPM-PZ-Cl (Supplementary Fig. 39)^56^. Meanwhile, the peak of Re 4f was observed at 46 eV in TFPM-PZ-Cl along with the disappearance of the Cl 2p peak in the XPS spectrum (197 eV), further indicating the ion exchange behavior^40^ (Supplementary Figs. 40 and 41). Furthermore, the peak of the N^+^ (−C − N^+^ − C −) increased from 401.21 to 401.71 eV after ReO_4_^-^ adsorbed (Supplementary Figs. 42 and 43), which were caused by the electrostatic attraction between the pyrazine group and the ReO_4_^−^^52^. EDS mapping further visually demonstrated the ion exchange behavior of Cl^−^ and ReO_4_^−^ (Fig. 5).

**Fig. 6:**
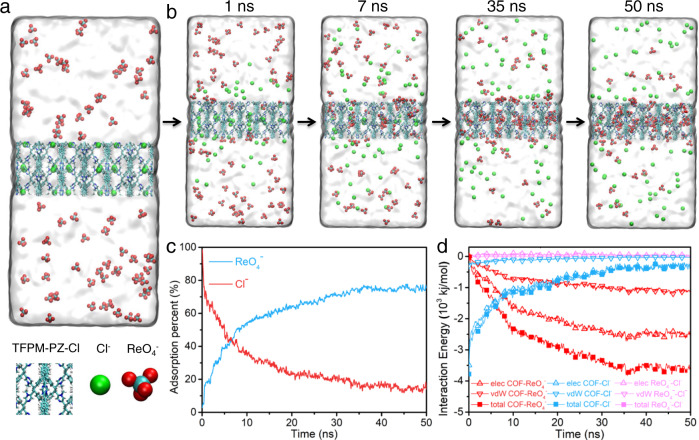
Kinetic simulation on the adsorption of ReO_4_^−^. **a** Kinetic simulation initial configuration (0 ns), water with a transparent surface. **b** Time series snapshot showing the ion exchange process. **c** Time evolution of the anion exchange rate of ReO_4_^−^ (blue) and the residence rate of Cl^−^ (red) in TFPM-PZ-Cl. **d** Variation of nonbonding interaction energies of COF- ReO_4_^−^ (red), COF-Cl^−^ (blue), and ReO_4_^−^-Cl^−^ (pink) with time; vdW represents van der Waals interaction; and elec represents electrostatic interaction.

We further explored the adsorption kinetics of ReO_4_^−^ in the TFPM-PZ-Cl structure using molecular dynamics (MD) simulations (Fig. 6a)^55^. After placing the structure of TFPM-PZ-Cl into a bulk ReO_4_^−^ aqueous solution, a lot of ReO_4_^−^ diffused in the aqueous solution was adsorbed very quickly into the internal structure of TFPM-PZ-Cl along with many Cl^−^ being desorbed from the structure (Fig. 6b). To objectively demonstrate this kinetic process, the adsorption rate of ReO_4_^−^ from the environment (Fig. 6c, blue curve) and the retention rate of pristine Cl^-^ (Fig. 6c, red curve) were analyzed. The kinetic curves showed that a very obvious intersects appeared at ca. 7 ns. After 7 ns, the number of ReO_4_^−^ anions adsorbed by TFPM-PZ-Cl exceeded the number of Cl^−^ anions remaining in TFPM-PZ-Cl. After about 35 ns, the ReO_4_^−^ adsorption rate almost unchanged and stabilized at ca. 75.7%. Meanwhile, the retention rate of Cl^−^ was ca. 13.8%. To reveal the driving forces of the adsorption process, the time evolution of the non-bonded interaction energies of COF-ReO_4_^−^, COF-Cl^−^, and ReO_4_^−^-Cl^−^ were computed, and they were further assigned to the electrostatic and van der Waals (vdW) interactions (Fig. 6d). In the time of the adsorption process of ReO_4_^−^ (<35 ns), the non-bonded interaction energy between ReO_4_^−^ anion and COF was reduced from 0 to −3600 kJ mol^−1^ (i.e., more favorable) (Fig. 6d, solid red square). Among them, the change of electrostatic part was about −2500 kJ mol^−1^ (Fig. 6d, open red triangle), which was about 2.3 times larger than that of the vdW part (−1100 kJ mol^−1^) (open red inverted triangle in Fig. 6d). Meanwhile, the nonbonding interaction between Cl^−^ and COF increased from −3800 to −300 kJ mol^−1^ (less favorable) (Fig. 6d, solid blue square), in which the electrostatic moiety contributed ~3200 kJ mol^−1^ (open blue triangles in Fig. 6d), and the vdW fraction contributed ~300 kJ mol^−1^ (open blue inverted triangles in Fig. 6d). Meanwhile, the non-bonded interaction energy between the Cl^-^ and ReO_4_^−^ anions increased by ~62 kJ mol^−1^ (less favorable) (Fig. 6d, solid pink square) with the electrostatic part increased by 66 kJ mol^−1^ (repulsive) (Fig. 6d, hollow pink triangle), whereas the vdW part decreased by ~4 kJ mol^−1^ (attractive) (Fig. 6d, hollow pink inverted triangle). These results indicated that this ReO_4_^−^ anions uptake process was strongly driven by the strong direct nonbonding interactions (especially electrostatic interactions) between ReO_4_^−^ anions and TFPM-PZ-Cl. In addition, the direct electrostatic repulsion between ReO_4_^−^ anions and Cl^−^ anions further promotes the adsorption of ReO_4_^−^ anions^55^.

In addition, we performed density functional theory (DFT) calculations to further understand the selective adsorption of ReO_4_^−^ by TFPM-PZ-Cl^57^. A typical TFPM-PZ-Cl fragment was used as the theoretical model (named Z^+^) (Supplementary Fig. 44). It could be found that the electron density on vdW surface near the pyrazine ring exhibited a relatively concentrated positive electrostatic potential (ESP) distribution (Supplementary Fig. 45)^40,54^, which laid the foundation for the good ion exchange behavior of the material. In addition, the calculated binding energies of ReO_4_^−^ was −68.68 kJ/mol, which was significantly higher than those of NO_3_^−^ (−38.71 kJ/mol) and SO_4_^2–^ (−13.96 kJ/mol). This can nicely explain why TFPM-PZ-Cl had such good selectivity to TcO_4_^−^/ReO_4_^−^ (see ref. 58).

## Discussion

In conclusion, we have synthesized ionic vinylene-linked 3D COFs (TAPM-PZI and TFPM-PZI) through the Aldol condensation. These crystalline frameworks showed regular pores and high chemical stability. In addition, the removal effect of TFPM-PZI as an ion exchange material for radionuclides (ReO_4_^−^/TcO_4_^−^) was further investigated. The results indicated that TFPM-PZI has rapid adsorption kinetics and good selectivity for radionuclide nuclear waste model ions (ReO_4_^−^). Our work not only broadens the category of 3D COFs but also promotes the application of COFs as efficient functional materials.

## Methods

### Synthesis of ionic 3D sp^2^ carbon-linked COFs

A 10-mL tube was charged with 3,5,7-tetrakis(4-aldophenyl)-adamantane (TAPM, 13.82 mg, 0.025 mmol) or tetrakis(4-formylphenyl)methane (TFPM, 11.03 mg, 0.025 mmol), 1,2,5-trimethylpyrazin-1-ium iodide (PZI, 12.75 mg, 0.050 mmol), 0.67 mL mesitylene, 0.67 mL 1,4-dioxane, 0.30 mL trifluoroacetic acid, and 0.037 mL acetonitrile. The mixture was degassed by three freeze–pump–thaw cycles, sealed under vacuum, and sonicated to yield a homogeneous solution. The reaction was heated in a 150 °C for 72 h. After the reaction completed, a deep brown colored powder was collected by centrifugation, washed several times with acetone, methanol and 0.1 mol L^−1^ NH_4_OH solution in aqueous methanol (50 wt%), respectively, and then washed with methanol in a Soxhlet extractor for 12 h. Finally, the material was dried and degassed at 120 °C under dynamic vacuum to 30 mTorr to yield TAPM-PZI (66.3%) and TFPM-PZI (73.4%) as a brown powder.

### Sorption kinetics study

The sorption kinetics experiments were performed under the conditions of pH 7 and solid–liquid ratio 0.5 g L^−1^. In total, 10 mg of TFPM-PZI was added into 20 mL aqueous solutions of the primal concentrations of ReO_4_^−^ (28 mg L^−1^). Under magnetic stirring, the resulting mixture was stirred for a required contact time, and then took 0.5 mL samples with using a 0.22-μm nylon membrane filter for ICP-MS detection. For the sorption kinetics experiments, the adsorption isotherms study of TFPM-PZI was performed by adding 5 mg TFPM-PZ-Cl into 10 mL aqueous solutions of the different initial concentrations of ReO_4_^−^ (*ca*. 50–670 mg L^−1^), then stirred overnight to reach equilibrium. The suspension was divided with a 0.22-µm nylon membrane filter for ICP-MS analysis.

## Supplementary information

Supplementary Information

## Data Availability

The data that support the findings of this study are available in the paper and its supplementary information files, or available from the corresponding author upon request.
