# Supplementary material for: An ionic vinylene-linked three-dimensional covalent organic framework for selective and efficient trapping of ReO4− or 99TcO4−
Source: Nat Commun. 2022 Dec 9;13:7621. doi: 10.1038/s41467-022-35435-7 (PMC9734744; doi:10.1038/s41467-022-35435-7)
Supplement: Supplementary file 1 — Supplementary Information [file 41467_2022_35435_MOESM1_ESM.pdf]

Supplementary Information

An ionic vinylene-linked three-dimensional covalent organic framework for selective and efficient trapping of ReO_4^- or $^{99}TcO_4^-$

Cheng-Rong Zhang,[1] Wei-Rong Cui,[1] Shun-Mo Yi,[1] Cheng-Peng Niu,[1] Ru-Ping Liang[1], Jia-Xin Qi,[1] Xiao-Juan Chen,[1] Wei Jiang,[1] Xin Liu,[1] Qiu-Xia Luo[1] & Jian-Ding Qiu[1,2]*

[1]College of Chemistry and Chemical Engineering, Nanchang University, Nanchang 330031, China

[2]State Key Laboratory of Nuclear Resources and Environment, East China University of Technology, Nanchang 330013, China

*Correspondence and requests for materials should be addressed to J.-D. Q. (email: jdqiu@ncu.edu.cn)

This PDF file includes:

Materials and Methods

Supplementary Figures 1 to 47

Supplementary Tables 1 to 8

Supplementary References 1-21

1. Supporting Experimental Procedures Section

Instruments. Fourier-transform infrared (FT-IR) spectra were recorded on a Nicolet Impact 410 FT-IR spectrometer. Powder X-ray diffraction (PXRD) data of the nanomaterials were collected on a Bruker AXS D8 Advance A25 Powder X-ray diffractometer (40 kV, 40 mA) using Cu Kα (λ=1.5406 Å) radiation. The morphology of the material was imaged by a scanning electron microscope (SEM, JEM-2010, JEOL). X-ray photoelectron spectroscopy (XPS) spectra were performed on a Thermo VG Multilab 2000X with Al Kα irradiation. The radiation stabilities of COFs were investigated in a GAMMATOR M-38-2 (USA) irradiator with a ^{60}Co source (γ-ray). The hydrophilic property of the COFs was observed on a contact angle measuring instrument (JY-82B Kruss DSA). Solid-state ^{13}C cross-polarization magic-angle spinning (^{13}C CP/MAS NMR) spectra were recorded with a 4-mm double-resonance MAS probe; a sample spinning rate of 10.0 kHz, a contact time of 2 ms (ramp 100), and a pulse delay of 3 s were applied. The nitrogen adsorption and desorption isotherms were measured at 77 K using a Micromeritics ASAP 2020M system. The samples were outgassed at 120 ℃ for 12 h before the measurements. Surface areas were calculated from the adsorption data using Brunauer-Emmett-Teller (BET) methods. The pore-size-distribution curves were obtained via the non-local density functional theory (NLDFT) method. The thermal properties of the nanomaterials were evaluated using a STA PT1600 Linseis thermogravimetric analysis (TGA) instrument over the temperature range of 30 to 800 ℃ under nitrogen atmosphere with a heating rate of 10 ℃/min. Metal ions concentrations were determined using an iCAP Q inductively coupled plasma mass spectrometry (ICP-MS, Thermo Fisher Scientific, USA). The light-absorbing property of samples is measured by an UV−vis−NIR spectrophotometer (LAMBDA950). Transmission electron microscope (TEM) were

performed on a JEOL JEM-F200 electron microscope with an accelerating voltage of 200 kV.**Materials and chemicals.** All reagents were obtained from commercial sources and used as received. The Re ICP standard solution (1000 mg/L in 2% nitric acid) was purchased from Henan Wanjia R&D Center Co., Ltd. tetrakis(4-formylphenyl)methane (TFPM) and 1,2,5-trimethylpyrazin-1-ium iodide (PZI) were purchased from Jilin Chinese Academy of Sciences-Yanshen Technology Co., Ltd. Sodium Perrhenate ($NaReO_4$) was purchased from Energy Chemical Technology (Shanghai) Co., Ltd. Anhydrous 1-methyl-2-pyrrolidinone, N, N-dimethylformamide (DMF), dichloromethane, and acetonitrile were purchased from Sinopharm Chemical Reagent Co., Ltd. Ultrapure water was prepared from the Millipore system (18.25 MΩ·cm). All reagents were used without further purification, and all the experiments were conducted at room temperature.

Stability test. TFPM-PZI wes exposed to the boiling water, γ-ray irradiation (200 kGy), NaOH (6.0 M), and HCl (6.0 M) for 48 h, respectively. The mixture was then filtered and washed with ultra-pure water till the supernatant became neutral and dried under vacuum at 60 ℃. Then, the FT-IR spectra and PXRD patterns were obtained.

Sorption isotherm study. The adsorption isotherms study of TFPM-PZI was conducted by adding 5 mg of adsorbent into 10 mL aqueous solutions of varying the initial concentrations of ReO_4^- (*ca.* 50-670 mg L^{-1}), then stirred overnight to achieve equilibrium. The suspension was separated with a 0.22 μm nylon membrane filter for ICP-MS analysis. The adsorption capacity was calculated based on equation (1).

$$q_e = \frac{(C_0 - Ce) \times V}{m} \qquad (1)$$

Where q_e (mg g^{-1}) is the equilibrium adsorption capacity, C_0 (mg L^{-1}) and C_e (mg L^{-1}) is the initial concentration and equilibrium concentration of ReO_4^-, respectively. *m* (mg) is the mass of the adsorbent and *V* (L) is the volume of the solution.

The Langmuir isotherm is based on the assumption that the adsorbent can only be adsorbed in a single layer on the adsorbent. The linear fitting of the Langmuir isotherm model is expressed by equation (2).

$$\frac{C_e}{q_e} = \frac{1}{q_m + k_L} + \frac{C_e}{q_m} \qquad (2)$$

where q_m (mg g^{-1}) is the maximum sorption capacity, k_L is a constant indirectly related to sorption capacity and energy of sorption (L mg^{-1}), which characterizes the affinity of the adsorbate with the adsorbent.

The Freundlich model is an empirical equation based on sorption on a heterogeneous surface. The linear fitting of the Freundlich isotherm model is expressed by equation (3).

$$\ln q_e = \ln k_F + \frac{1}{\mathrm{n}} \ln C_e \qquad (3)$$

where k_F and n are the Freundlich constants related to the sorption capacity and the sorption intensity, respectively.

Sorption kinetics study. The sorption kinetics experiments were carried out under the conditions of pH 7 and solid-liquid ratio 0.5 g L^{-1}. 10 mg of TFPM-PZI was added into 20 mL aqueous solutions of the initial concentrations of ReO_4^- (28 mg L^{-1}). Under magnetic stirring, the resulting mixture was stirred for a desired contact time (30 s, 1 min, 2 min, 5 min, 10 min, 20 min, 40 min and 60 min), then take 0.5 mL samples and using 0.22 μm nylon membrane filter for ICP-MS detection. The removal rate (RE) was calculated based on equation (4).

$$\mathrm{RE} = \frac{C_0 - C_t}{C_0} \times 100\% \qquad (4)$$

where C_t is the concentration of ReO_4^- at time t.

Pseudo first-order model and pseudo-second-order model are usually used for the sorption kinetics data fitting. The formulas were expressed in equations (5) and (6), respectively.

$$\ln(q_e - q_t) = \ln q_e - k_f t \quad (5)$$

$$\frac{t}{q_t} = \frac{1}{k_s + q_e^2} + \frac{1}{q_e} t \quad (6)$$

where q_e and q_t are the adsorption capacity at equilibrium and time t, k_1 (g mg^{-1} min^{-1}) and k_2 (g mg^{-1} min^{-1}) are the rate constants of pseudo-first-order and pseudo-second-order, respectively. The pseudo-first-order linear plot can be obtained by plotting ln (q_e-q_t) versus t, and the pseudo-second-order linear plot can be obtained by plotting t/q_t against.

Anion selectivity study. 10 mg TFPM-PZI was added to 10 mL 0.1 mM ReO_4^- aqueous solution, which contained 0.1 mM of the competing anions, SO_4^{2-}, NO_3^-, PO_4^{3-}, and CO_3^{2-} respectively. 10 mg adsorbent was added to aqueous solution containing 0.1 mM ReO_4^-, following the different concentration of $NaNO_3$ (0.1 mM, 1 mM, 10 mM, 50 mM) or Na_2SO_4 (0.1 mM, 1 mM, 10 mM, and 100 mM) was mixed into the solution, and the resulting were shaken at a rate of 150 rpm for 12 h, Then using a 0.22 μm nylon membrane filter for ICP-MS detection.

pH optimization. 10 mg adsorption material was added to 20 mL aqueous solution containing 28 ppm ReO_4^- with varying pH from 2 to 10, and the solution pH was adjusted as required using NaOH and HNO_3. The mixtures were shaken at a rate of 150 rpm for 12 h, and separated with a 0.22 μm nylon membrane filter for ICP analysis.

Removal rate in simulated wastes. According to reported research, we prepared the simulated Hanford LAW Melter Recycle solution. The experiment was

conducted as follows. 10 mg, 50 mg TFPM-PZI was added to 10 mL of the above simulated solution, respectively. After being shaken at a rate of 150 rpm for 24 h, the mixtures were separated by using a 0.22 μm nylon membrane filter for the ICP-MS test.

Recyclability test. The recyclability experiments were conducted using a solution containing 28 mg L^{-1} ReO_4^- at pH 7 and the solid-liquid ratio is 1 g L^{-1}. After one run of adsorption, the TFPM-PZ-ReO_4^- was regenerate by 3 M NaCl at 80 ℃.

Electrochemical studies. Indium-tin oxide (ITO) glasses were firstly cleaned by sonication in ethanol for 30 min and dried under nitrogen flow. 5 mg of COF powder was mixed with 1 mL *n*-BuOH and ultra-sonicated for 30 min to get slurry. 150 μL of the slurry was spreading onto ITO glass. After air drying, the boundary of the electrode was isolated with epoxy resin. A conventional three electrodes cell was used with a platinum mesh as the counter electrode and an Ag/AgCl electrode (saturated KCl) as reference electrode. The electrolyte was a 5 mM $K_3[Fe(CN)_6]$ aqueous solution and was purged with nitrogen gas for 1 h prior to the measurements. The working electrodes were immersed in the electrolyte for 60 s before any measurements were taken. The photocurrent responses were conducted with a CHI 760E workstation, with the working electrodes irradiated from the front side. The light was generated by a 300W xenon lamp with a light density of 1 kW m^{-2} at room temperature with the light wavelength from 300 nm to 2500 nm.

Molecular dynamics simulation: Atomistic molecular dynamics (MD) simulations have been performed in the GROMACS[1] (version 2020.6) simulation package using OPLSAAM force field[2-4]. The COF material with 3 × 3 × 2 repeat units of around 7.2× 7.2 × 2.7 nm, which contain 5904 atoms including 72 CL^- ions, was constructed through the Materials Studio simulation software. An infinite membrane system was

modelled with 2D periodic bonding with its images while expanding the size normal to the surface to accommodate water molecules. The parameters for the Re atom used the Universal force field (UFF)[5], which covers the whole periodic table and the charges adopted the DFT calculations using the B3LYP/LANL2DZ method. $ReO4^-$ ions were randomly inserted into both sides of the COF surface before solvation of more than 20000 water molecules using the TIP4P water model. After thousands steps of energy minimization, the iso-thermo and iso-baric (NPT) ensemble MD run of 5 ns were performed to equilibrate the system. Finally, the production runs were performed under the constant volume canonical ensemble (NVT) using the nose-hoover temperature coupling method at 298 K for another 50 ns. A cutoff length of 1.2 nm was implemented for the non-bonded interactions, and the Particle Mesh Ewald method[6] with a fourierspacing of 0.1 nm was applied for the long range electrostatic interactions. All covalent bonds with hydrogen atoms were constraint using the LINCS algorithm[7].

DFT calculation detail. A fragment (Z^+ for short in the following, as shown in Supplementary Figure 44) was used as the model for mimicking the positively charged local structure of the amorphous sorbent material of TFPM-PZI. Four anions, including TcO_4^-/ReO_4^-, NO_3^- and SO_4^{2-} were used as the sorbates. The structure optimization, and energy of the ground state involved in the calculation are all calculated by used the Gaussian 16 program. Select B3LYP for functional, 6-31g(d) for non-metallic element basis and lanl2dz for Re, and "Tight" for the self-consistent field (SCF) and structural optimization convergence criteria, and 6-311g (d,p)/SDD for energy calculation. The density functional dispersion correction (DFT-D3) method of Grimme with Becke-Johnson damping was adopted to describe the weak bound molecular systems. The SMD implicit solvent model was employed to consider the

solvent effect. Based on the single-point energy calculations, the binding energies, Eb, were calculated based on equation (7).

$$E_b = E_{\text{complex}} - E_{z+} - E_{\text{anion}} \qquad (7)$$

where E(complex), E(Z^+), and E(anion) indicate the total energies of each complex, Z^+, and anions, respectively. All calculations were performed using the Gaussian 09 program 69.

Synthesis of Model compound (A). Molecular model compound (A) was synthesized under similar reaction conditions to those of TFPM-PZI. A Pyrex tube measuring 10 × 8 mm (o.d. × i.d.) was charged with 1,2,5-trimethylpyrazin-1-ium iodide (PZI, 9.37 mg, 0.0375 mmol), benzaldehyde (15.9 mg, 0.075 mmol), 0.45 mL mesitylene, 0.45 mL 1,4-dioxane, 0.20 mL trifluoroacetic acid, and 0.025 mL acetonitrile. The tube was flash frozen at 77 K (liquid N_2 bath), evacuated to an internal pressure of 500 mTorr, and flame sealed. Upon sealing, the length of the tube was reduced to 7−8 cm. The reaction was carried out at 150 °C for 24 h to give a red solution. The solution was further neutralized with saturated aqueous $NaHCO_3$ solution and evaporated under reduced pressure to remove volatile compounds. The resulting solid was extracted with dichloromethane (DCM, 50 mL), washed with water (50 mL) and brine (50 mL), and dried over Na_2SO_4. The solvent was evaporated, and the product was purified by flash column chromatography (hexanes/DCM = 1:4) to yield an orange solid.

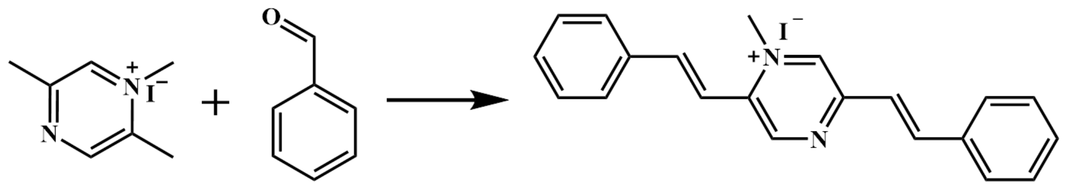

2. Supporting Results and Discussion Section

Supplementary Table 1 | Reaction conditions with different catalysts for the synthesis of TAPM-PZI.

Conditions	Ratio	Temperature	Catalyst	Product
mesitylene/1,4-dioxane	1/1	150 ℃	Trifluoroacetic acid	High crystallinity
nBuOH/o-DCB	7/3	120 ℃	MeONa	Amorphous polymer
nBuOH/o-DCB	7/3	120 ℃	EtONa	Amorphous polymer
nBuOH/o-DCB	7/3	120 ℃	KOH	Amorphous polymer
o-DCB	1	100 ℃	EtONa	Amorphous polymer
nBuOH	1	100 ℃	EtONa	Amorphous polymer
o-DCB/Ethanol	6/4	100 ℃	KOH	Amorphous polymer

Supplementary Table 2 | Reaction conditions with different catalysts for the synthesis of TFPM-PZI.

Conditions	Ratio	Temperature	Catalyst	Product
mesitylene/1,4-dioxane	1/1	150 ℃	Trifluoroacetic acid	High crystallinity
nBuOH/o-DCB	7/3	120 ℃	MeONa	Amorphous polymer
nBuOH/o-DCB	7/3	120 ℃	EtONa	Amorphous polymer
nBuOH/o-DCB	7/3	120 ℃	KOH	Amorphous polymer
o-DCB	1	100 ℃	EtONa	Amorphous polymer
nBuOH	1	100 ℃	EtONa	Amorphous polymer
o-DCB/Ethanol	6/4	100 ℃	KOH	Amorphous polymer
DMF	1	150 ℃	Pyridine	No product

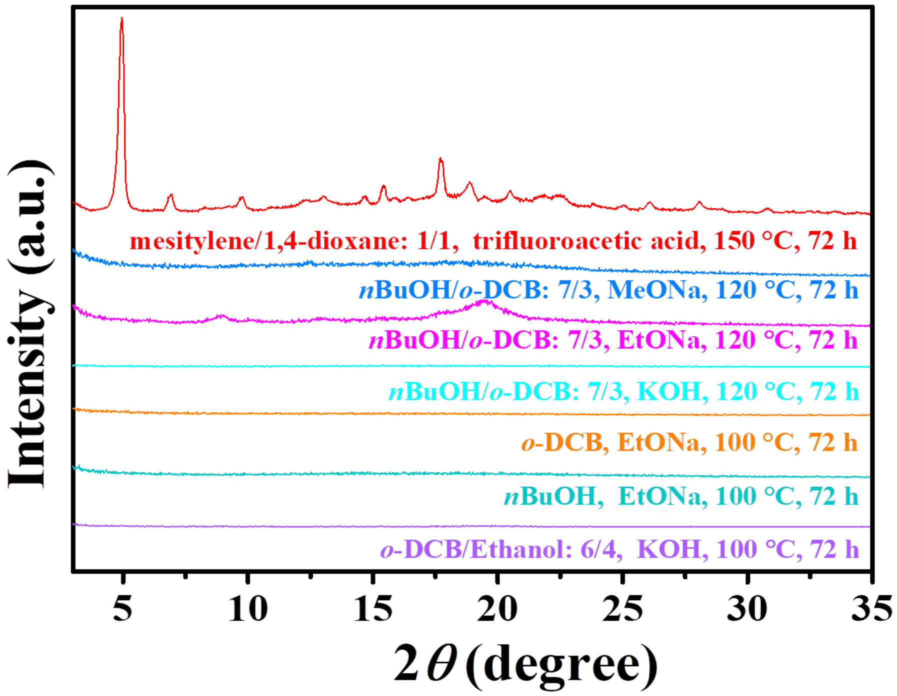

Supplementary Figure 1 | PXRD profiles of attempts to synthesize TAPM-PZI under different reaction conditions.

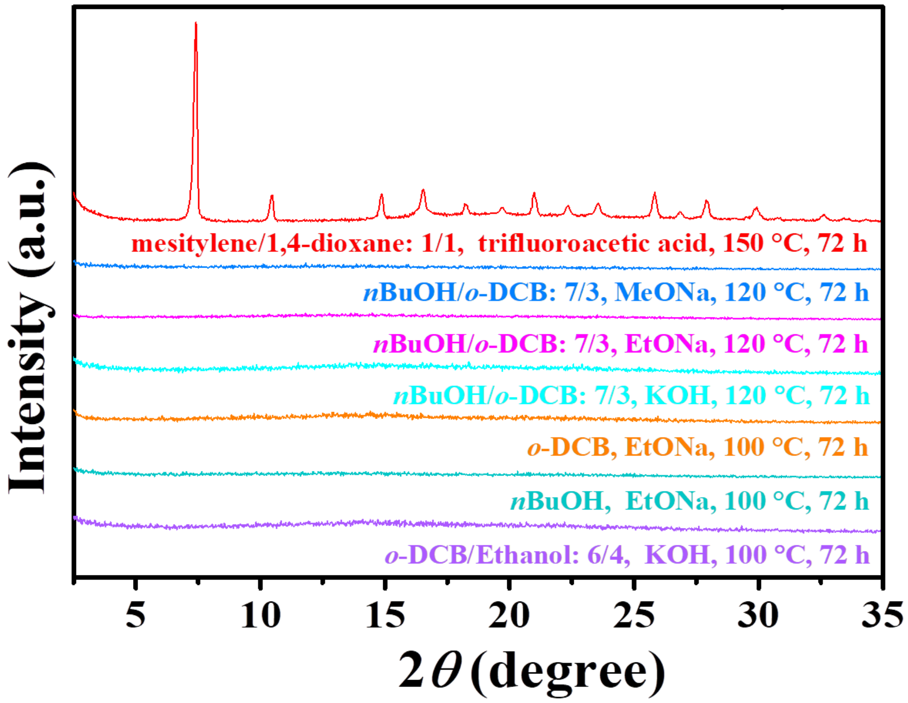

Supplementary Figure 2 | PXRD profiles of attempts to synthesize TFPM-PZI under different reaction conditions.

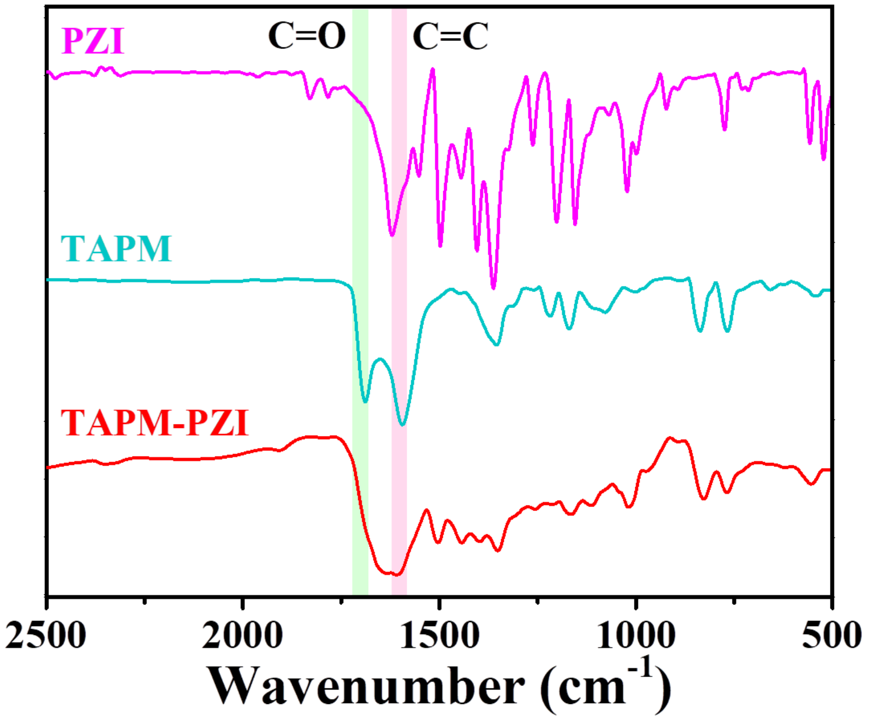

Supplementary Figure 3 | Fourier transform infrared spectra of PZI, TFAM and TAPM-PZI.

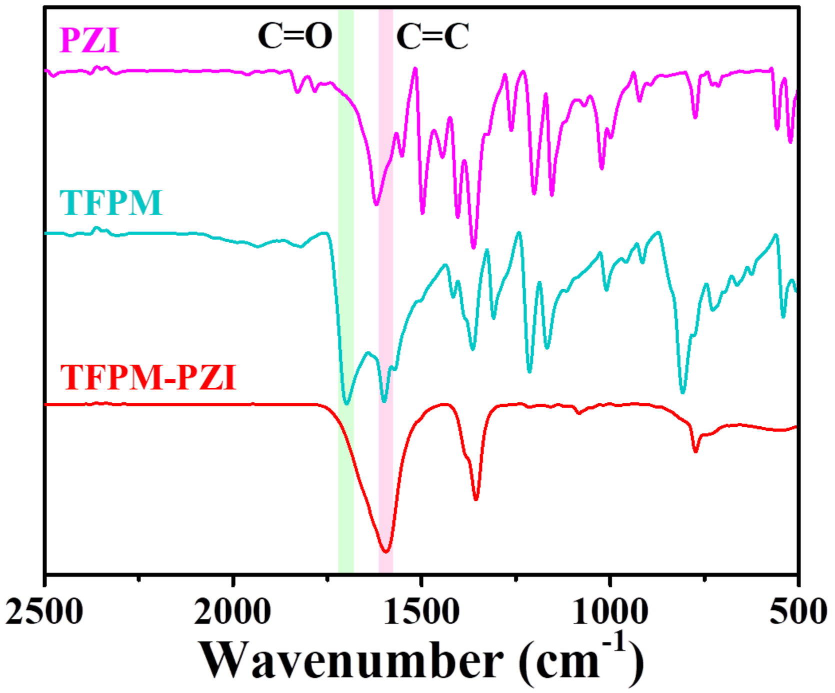

Supplementary Figure 4 | FT-IR spectra of PZI, TFPM and TFPM-PZI.

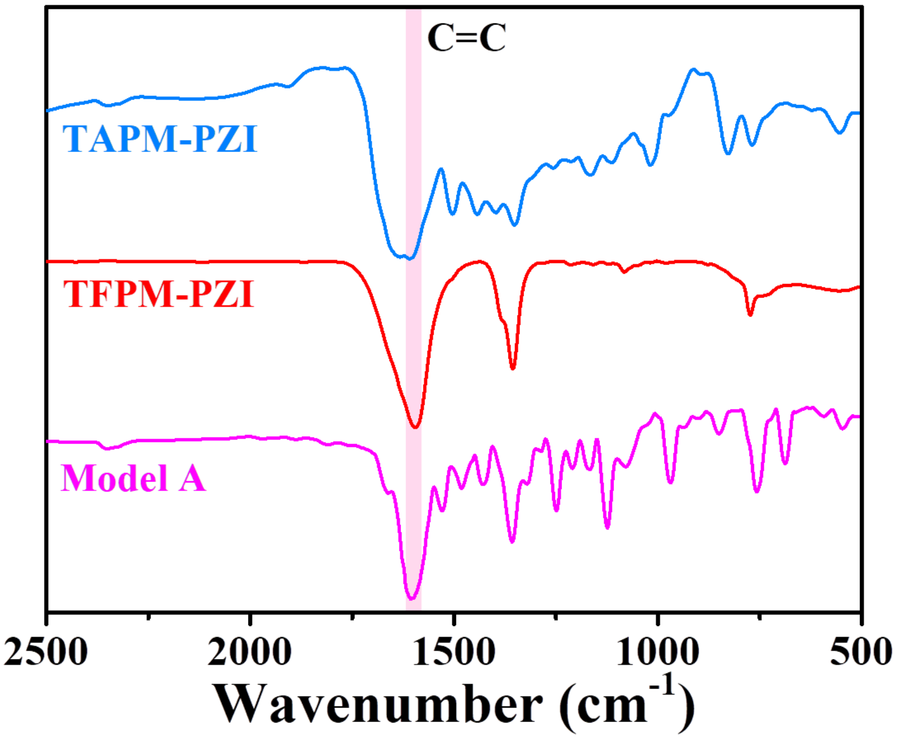

Supplementary Figure 5 | FT-IR spectra of TAPM-PZI, TFPM-PZI and Model A.

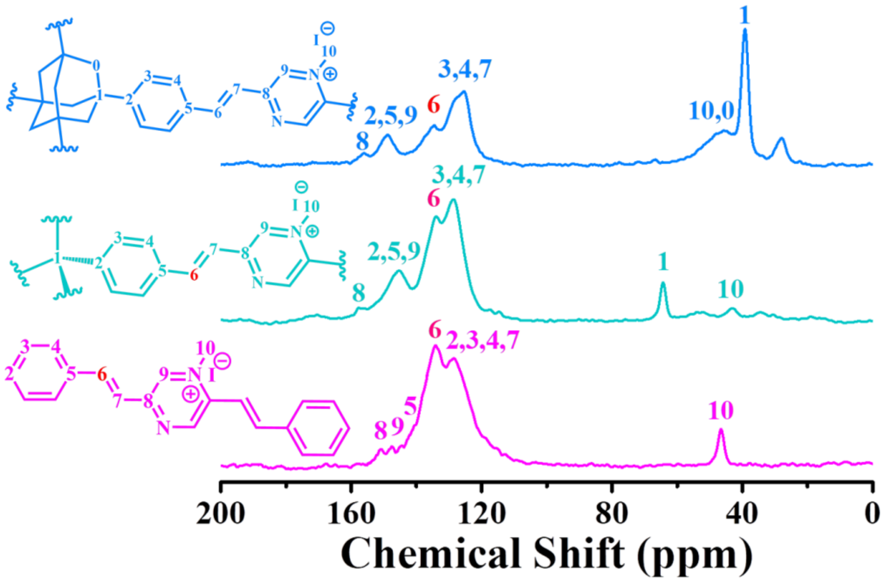

Supplementary Figure 6 | Solid-state ^{13}C cross-polarization/magic angle spinning (^{13}C CP-MAS) NMR spectrum of the TAPM-PZI, TFPM-PZI and Model A.

Supplementary Table 3 | Unit cell parameters and fractional atomic coordinates for TAPM-PZI calculated based on the 8-fold interpenetrated dia net.

**Space group: P-4 (No. 81)**<br>**a = 25.5427 b = 25.5427 c = 6.3857**<br>**alpha = 90.000 beta = 90.000 gamma = 90.000**							
Atom	*X*	*Y*	*Z*	*Atom*	*X*	*Y*	*Z*
C1	0.42366	0.43466	0.71831	C26	0.5036	0.56988	0.99821
C2	0.42162	0.37914	0.71572	C27	0.29138	0.79496	0.56014
C3	0.38582	0.35221	0.58832	H28	0.44758	0.35558	0.81003
C4	0.3502	0.37958	0.46189	H29	0.38558	0.30974	0.58972
C5	0.35138	0.4344	0.46442	H30	0.32423	0.45658	0.36891
C6	0.38748	0.46137	0.58943	H31	0.38697	0.50356	0.58443
C7	0.3111	0.35195	0.32944	H32	0.28149	0.3748	0.24901
N8	0.31243	0.30141	0.30513	H33	0.33313	0.24755	0.95771
C9	0.27508	0.27259	0.18158	H34	0.09702	1.01095	0.89667
C10	0.29235	0.24579	1.00269	H35	0.09098	0.96132	1.09537
N11	0.2234	0.26825	0.24178	H36	0.10293	0.98178	0.55336
C12	0.04056	0.02999	0.14174	H37	0.15555	0.9223	0.3362
C13	0.06881	0.98975	0.99842	H38	0.08172	0.79009	0.63121
C14	0.06098	0.92086	0.71758	H39	0.02962	0.84767	0.84474
C15	0.09745	0.94011	0.57038	H40	0.14873	0.77282	0.37165
C16	0.12762	0.90614	0.44619	H41	0.23429	0.85124	0.83209
C17	0.12213	0.85169	0.46531	H42	0.47973	0.5964	0.89864
C18	0.08636	0.83204	0.61179	H43	0.53002	0.59437	1.09507
C19	0.05628	0.86578	0.73555	H44	0.28005	0.76818	0.43019
C20	0.15308	0.81424	0.33751	H45	0.27758	0.83482	0.51634
N21	0.1834	0.82932	0.18467	H46	0.33476	0.79591	0.57189
C22	0.2132	0.79326	0.05848	H47	0.52398	0.47274	1.63741
C23	0.23886	0.81061	0.87709	I48	0.76084	0.24175	2.67207
N24	0.21749	0.74207	0.11422	C49	0	0	0.7239
C25	0.4662	0.53798	0.1397	C50	0.5	0.5	0.73061

Supplementary Table 4 | Unit cell parameters and fractional atomic coordinates for TFPM-PZI calculated based on the 5-fold interpenetrated dia net.

Space group: P-4 (No. 81)<br>a = 23.88942 b = 23.88942 c = 13.53205<br>alpha = 90.000 beta = 90.000 gamma = 90.000							
Atom	*X*	*Y*	*Z*	*Atom*	*X*	*Y*	*Z*
C1	0.4554	0.7621	-0.4457	C43	0.3892	1.0478	0.4466
C2	0.4934	0.7201	-0.4791	C44	0.4252	1.0477	0.3594
C3	0.4844	0.6889	-0.5819	C45	0.4482	0.9984	0.3150
N4	0.5360	0.7079	-0.4139	C46	0.4329	0.9460	0.3714
C5	0.5207	0.6531	-0.6319	C47	0.3979	0.9462	0.4585
C6	0.5132	0.6213	-0.7348	H48	0.2933	1.0302	0.5655
C7	0.5602	0.5954	-0.7809	H49	0.3724	1.0876	0.4712
C8	0.5566	0.5620	-0.8701	H50	0.4360	1.0878	0.3270
C9	0.5054	0.5515	-0.9214	H51	0.4497	0.9064	0.3460
C10	0.4581	0.5781	-0.8758	H52	0.3879	0.9068	0.4943
C11	0.4618	0.6123	-0.7864	H53	0.3697	0.9669	0.7203
H12	0.5608	0.6468	-0.5956	H54	0.3188	0.9239	0.8599
H13	0.6009	0.5998	-0.7457	C55	0.5420	0.7339	-0.3215
H14	0.5943	0.5416	-0.8962	C56	0.5042	0.7761	-0.2875
H15	0.4169	0.5702	-0.9066	C57	0.5097	0.7982	-0.1759
H16	0.4237	0.6297	-0.7556	N58	0.4608	0.7889	-0.3529
H17	0.4449	0.6972	-0.6190	C59	0.4969	0.8517	-0.1280
H18	0.4209	0.7727	-0.4949	C60	0.5032	0.8635	-0.0178
C19	0.2163	1.0449	0.8871	C61	0.4509	0.8841	0.0377
C20	0.2176	1.0036	0.9645	C62	0.4522	0.9217	0.1219
C21	0.1798	1.0115	1.0530	C63	0.5017	0.9480	0.1586
N22	0.2540	0.9614	0.9512	C64	0.5507	0.9369	0.1005
C23	0.1642	0.9712	1.1430	C65	0.5522	0.9005	0.0146
C24	0.1275	0.9854	1.2407	H66	0.4822	0.8857	-0.1745

C25	0.1190	0.9430	1.3143	H67	0.4108	0.8669	0.0155
C26	0.0828	0.9490	1.3997	H68	0.4126	0.9324	0.1562
C27	0.0521	0.9985	1.4214	H69	0.5887	0.9593	0.1191
C28	0.0611	1.0431	1.3510	H70	0.5894	0.8961	-0.0229
C29	0.0967	1.0383	1.2641	H71	0.5285	0.7680	-0.1254
H30	0.1460	0.9328	1.1095	H72	0.5761	0.7212	-0.2733
H31	0.1394	0.9030	1.3034	C73	0.4183	0.8301	-0.3241
H32	0.0770	0.9126	1.4468	C74	0.2594	0.9196	1.0237
H33	0.0380	1.0816	1.3607	H75	0.4011	0.8201	-0.2485
H34	0.1001	1.0734	1.2130	H76	0.4368	0.8726	-0.3243
H35	0.1547	1.0493	1.0525	H77	0.3824	0.8303	-0.3773
H36	0.1880	1.0801	0.8927	H78	0.2692	0.9383	1.0982
C37	0.2890	0.9579	0.8664	H79	0.2206	0.8942	1.0270
C38	0.2897	0.9984	0.7876	H80	0.2941	0.8900	1.0070
C39	0.3377	0.9992	0.7034	C82	0.5000	1.0000	0.2369
N40	0.2534	1.0417	0.8001	C83	0.5000	0.5000	-1.0000
C41	0.3257	0.9987	0.5845	C84	0.0000	1.0000	1.5000
C42	0.3745	0.9990	0.5020				

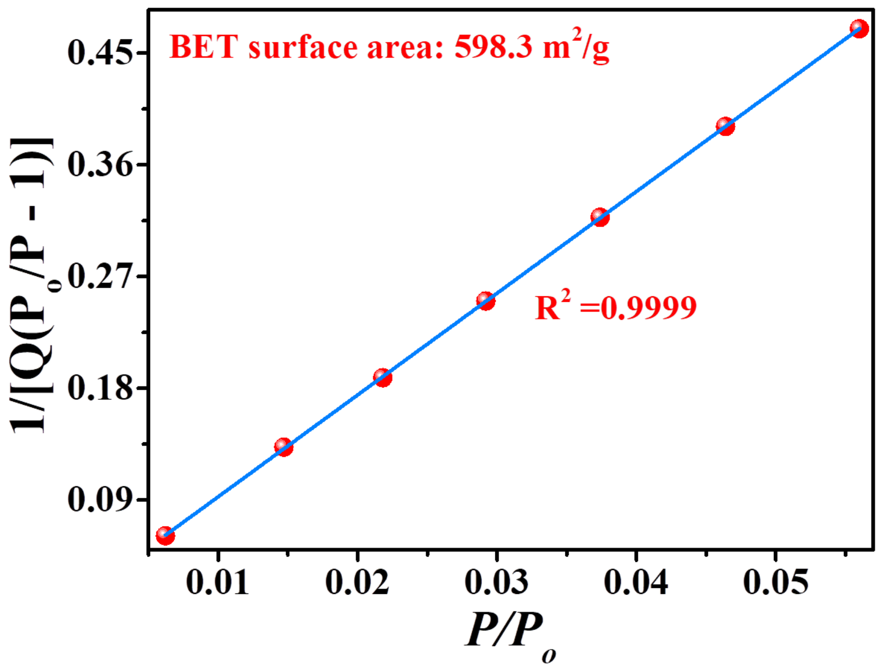

Supplementary Figure 7丨 BET surface area plots for TAPM-PZI calculated from the isotherms.

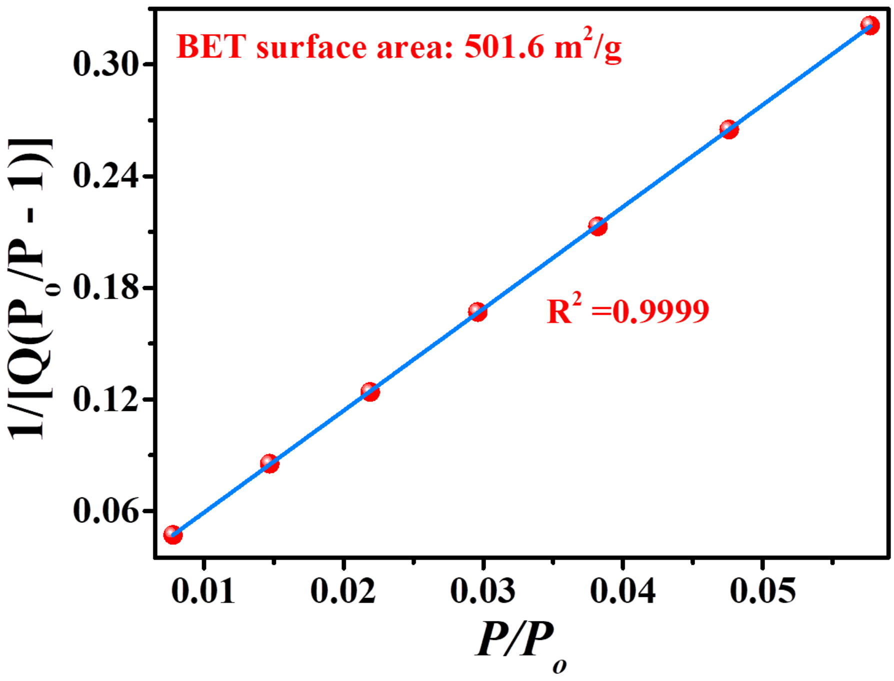

Supplementary Figure 8丨 BET surface area plots for TFPM-PZI calculated from the isotherms.

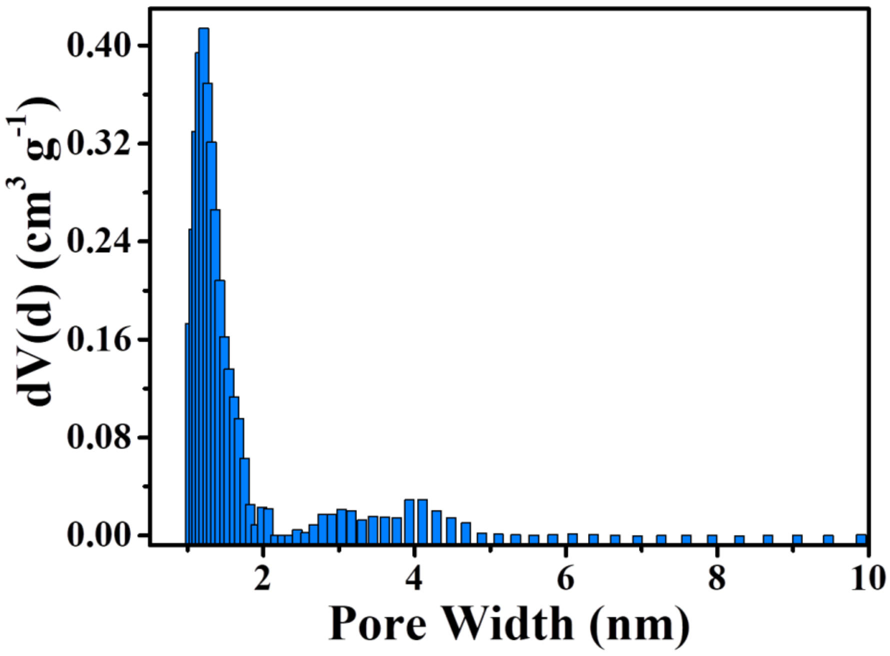

Supplementary Figure 9 | The pore-size distributions of TAPM-PZI calculated from non-local density functional theory.

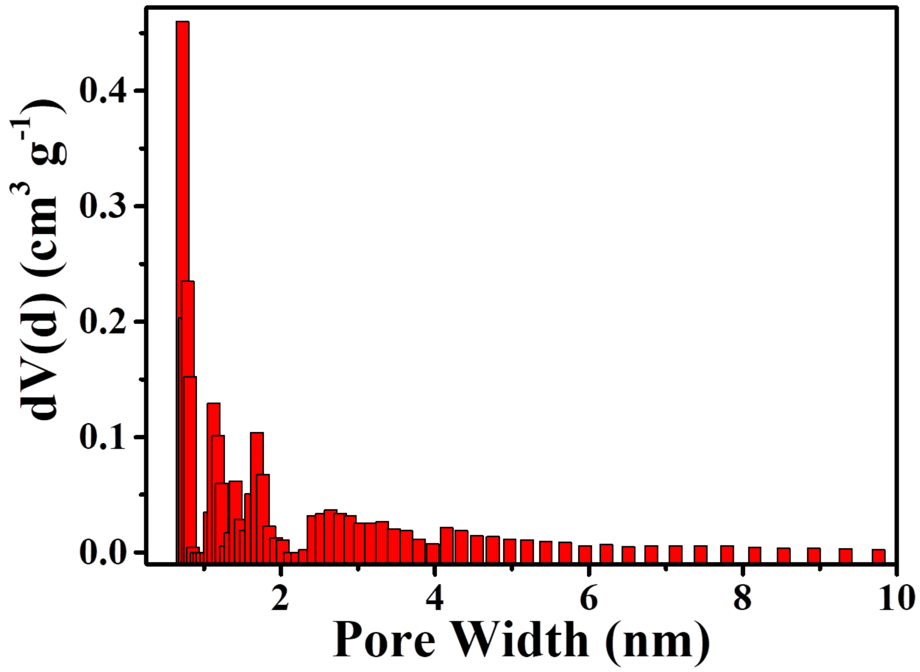

Supplementary Figure 10 | The pore-size distributions of TFPM-PZI calculated from non-local density functional theory.

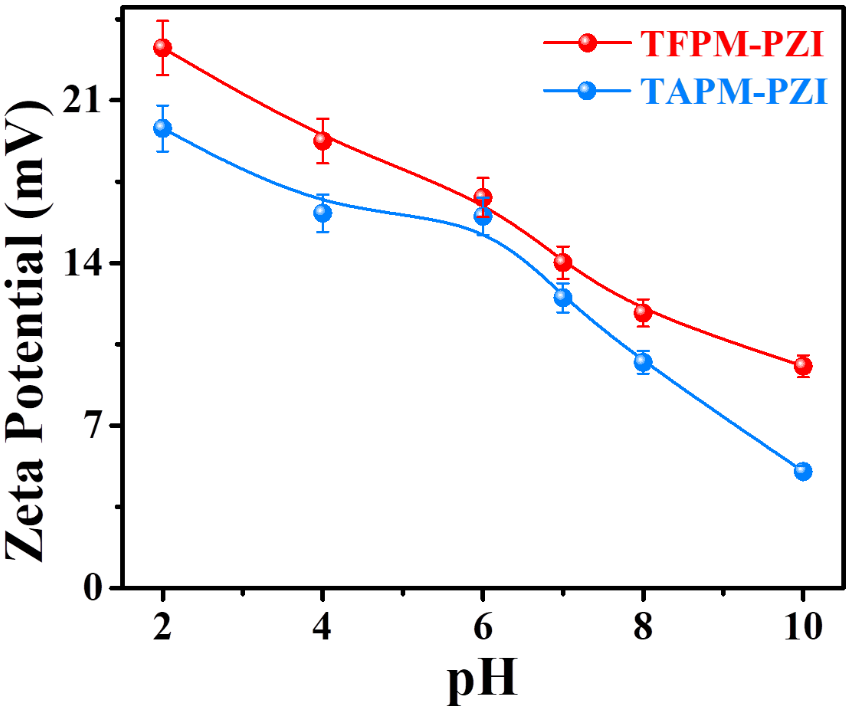

Supplementary Figure 11 | The Zeta potential analysis for TFPM-PZI and TAPM-PZI. Error bars represent S.D. n=3 independent experiments.

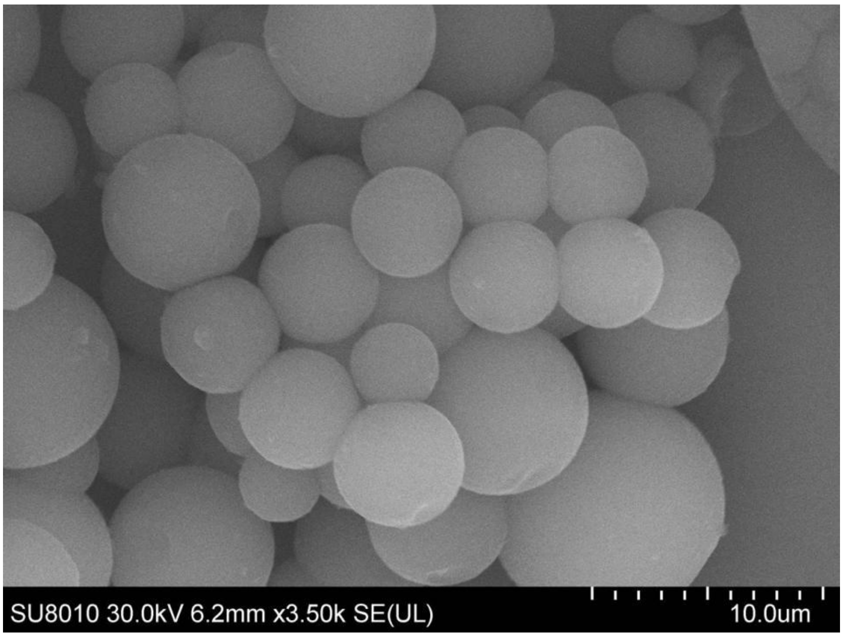

Supplementary Figure 12 | Scanning electron microscopy (SEM) image of TAPM-PZI.

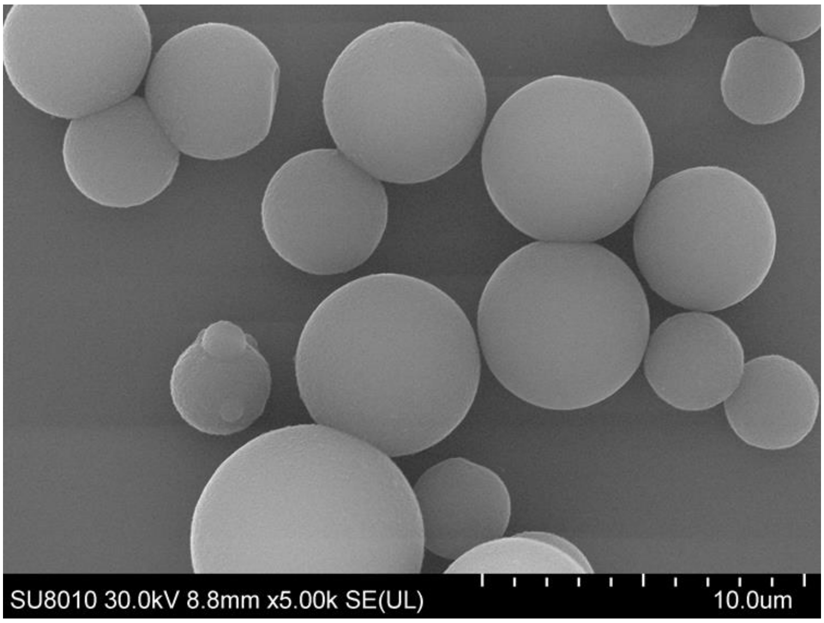

Supplementary Figure 13 | SEM image of TFPM-PZI.

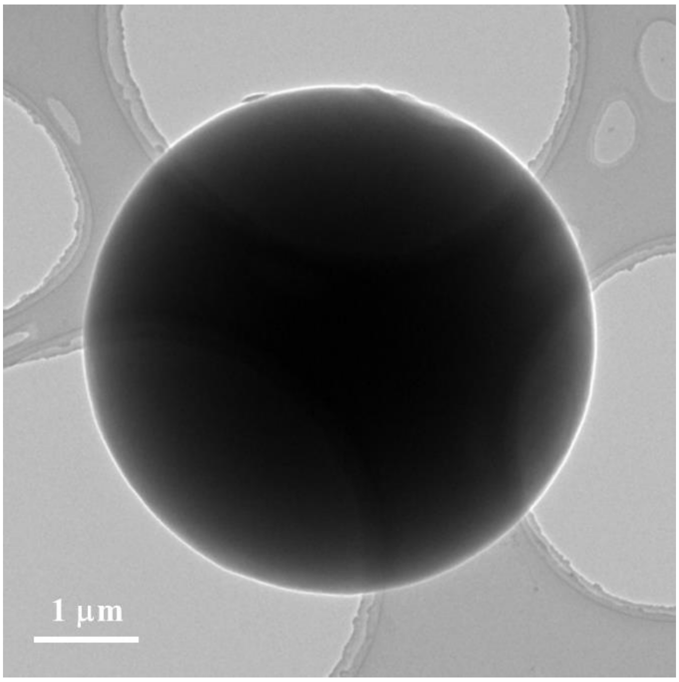

Supplementary Figure 14 | Transmission electron microscopy (TEM) image of TAPM-PZI.

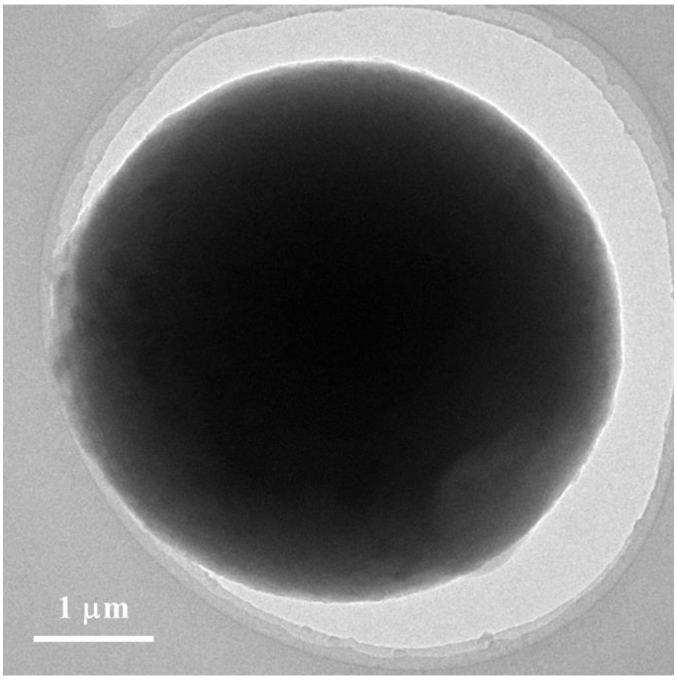

Supplementary Figure 15 | TEM image of TFPM-PZI.

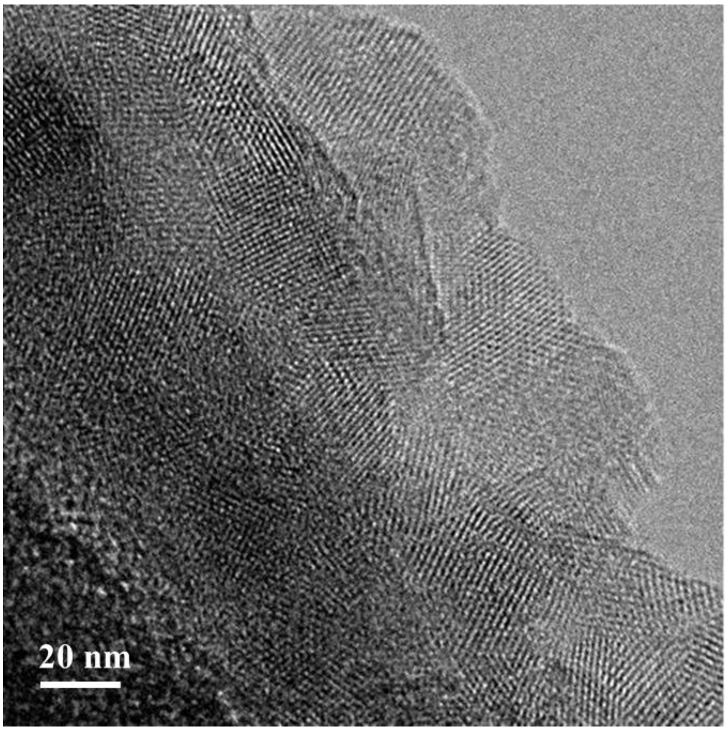

Supplementary Figure 16 | High resolution transmission electron microscope (HRTEM) image of TAPM-PZI.

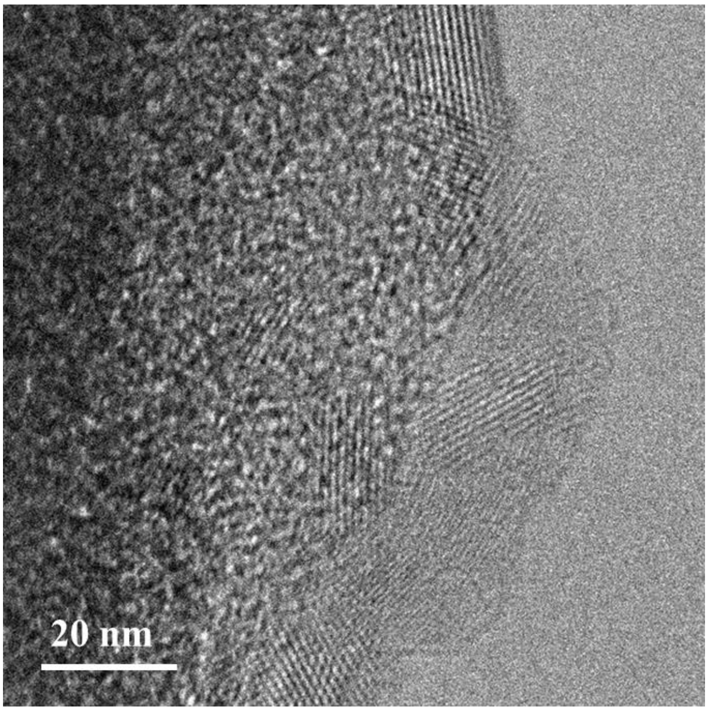

Supplementary Figure 17 | HRTEM image of TFPM-PZI.

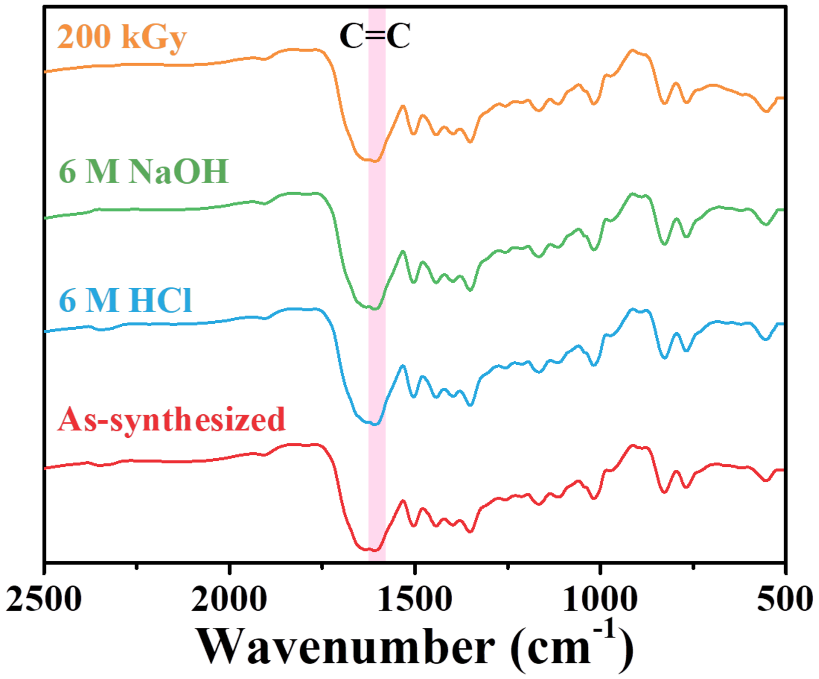

Supplementary Figure 18 | FT-IR spectra of TAPM-PZI before and after treatment with 200 kGy γ-ray irradiation, 6 M NaOH, 6 M HCl for 48 h.

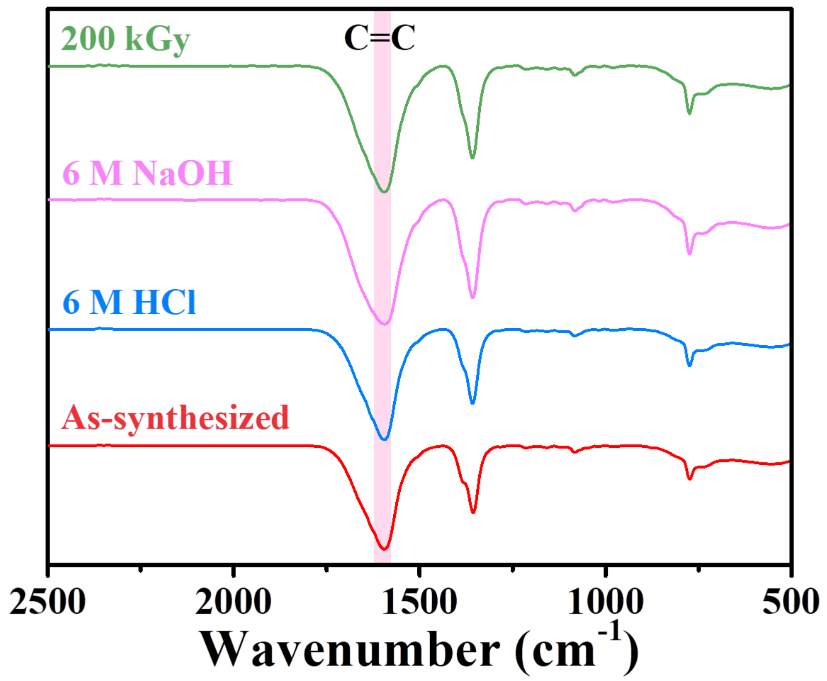

Supplementary Figure 19 | FT-IR spectra of TFPM-PZI before and after treatment with 200 kGy γ-ray irradiation, 6 M NaOH, 6 M HCl for 48 h.

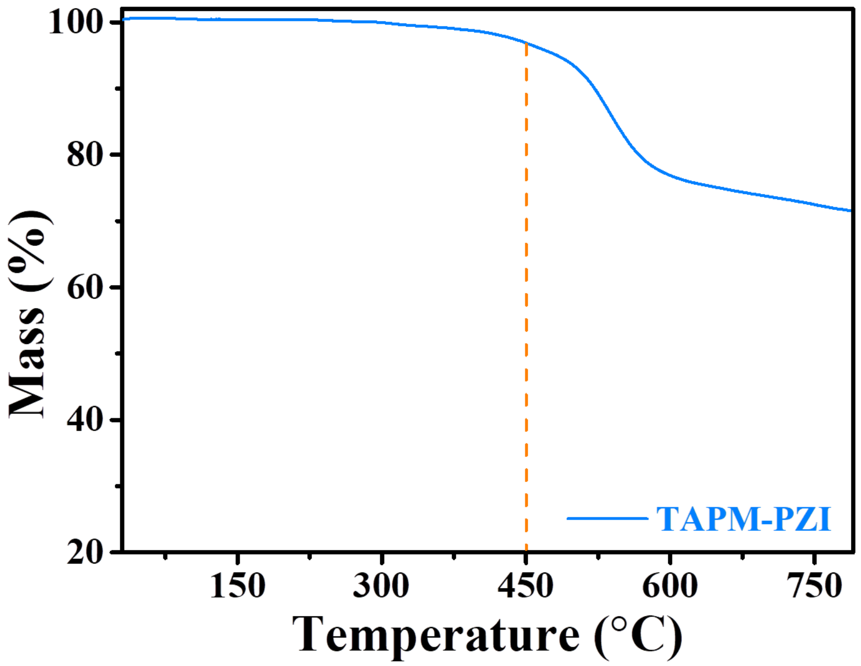

Supplementary Figure 20 | Thermogravimetric analysis (TGA) curve of the TAPM-PZI under nitrogen.

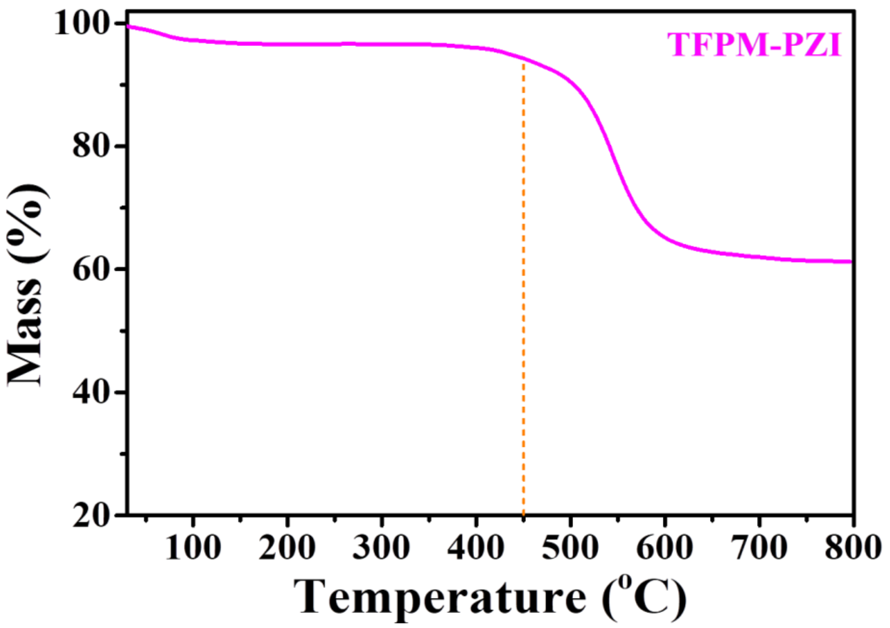

Supplementary Figure 21 | TGA curve of the TFPM-PZI under nitrogen.

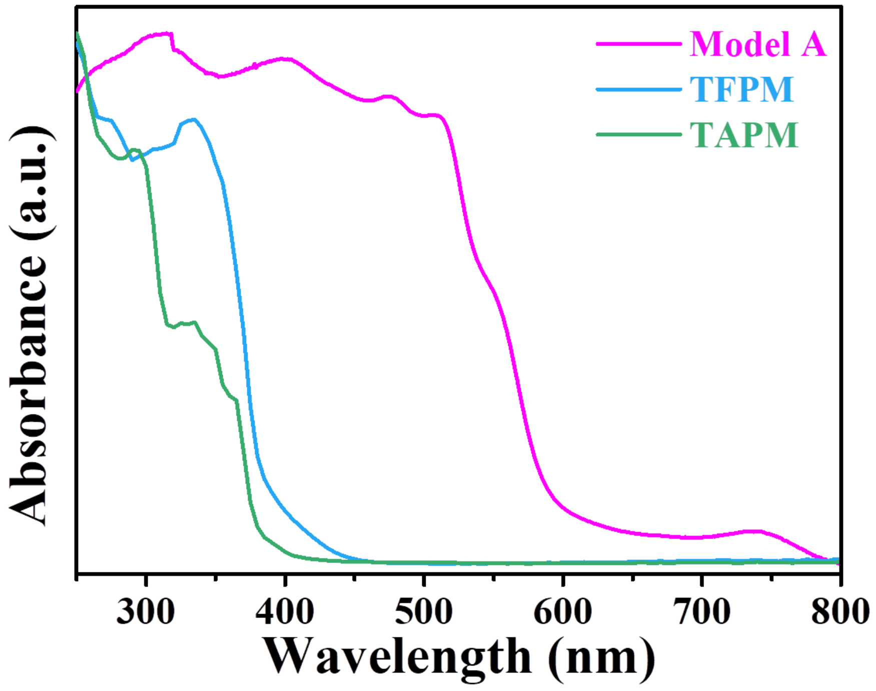

Supplementary Figure 22 | UV/Vis DRS spectrum of Model A, TFPM and TAPM.

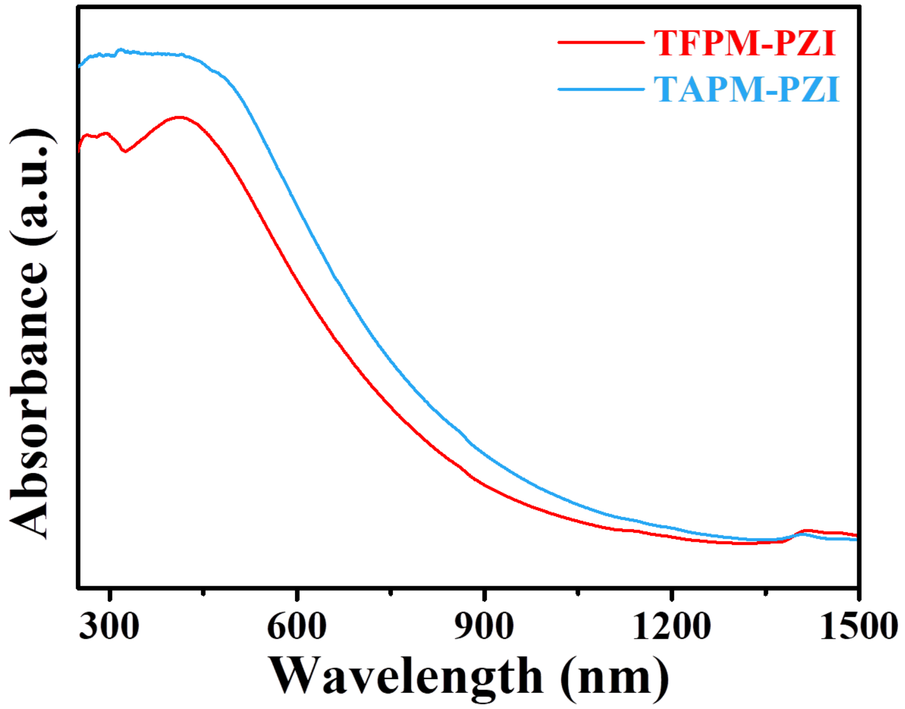

Supplementary Figure 23 | UV/Vis DRS spectrum of TAPM-PZI and TPFM-PZI.

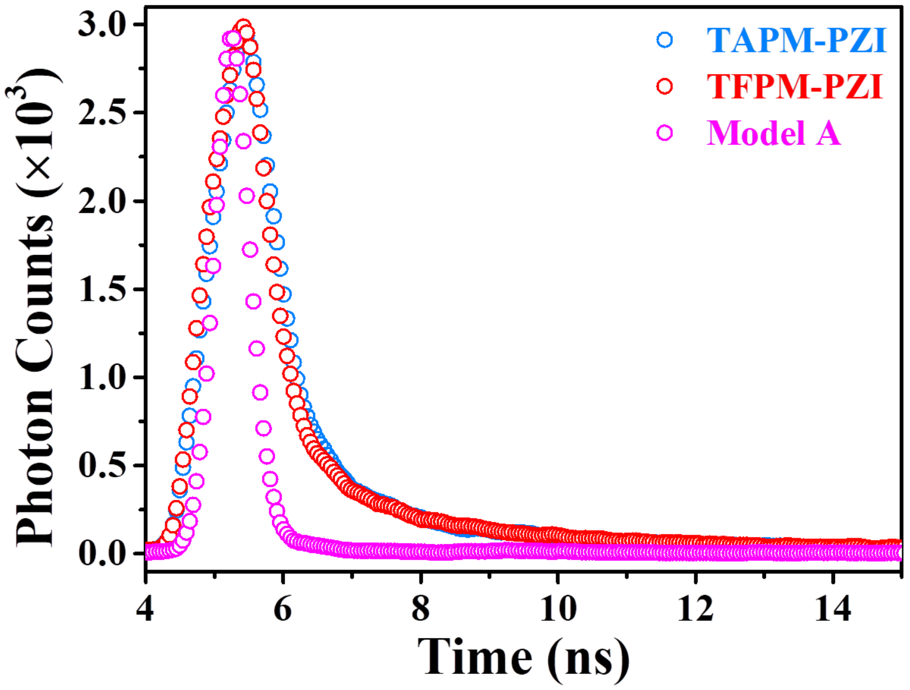

Supplementary Figure 24 | Fluorescence decay spectra of TAPM-PZI, TFPM-PZI and Model A.

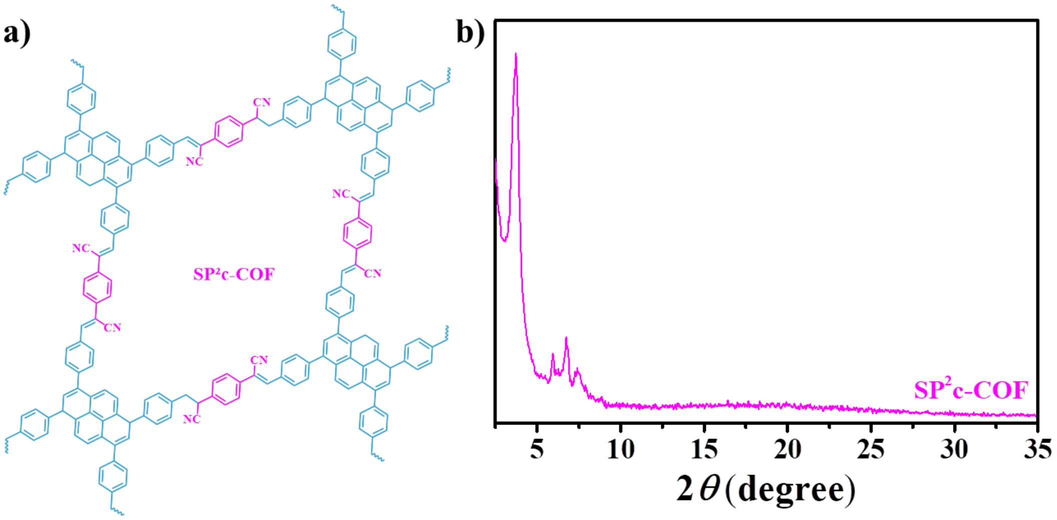

Supplementary Figure 25 | (a) The structure of SP^{2}c-COF. (b) PXRD pattern of SP^{2}c-COF.

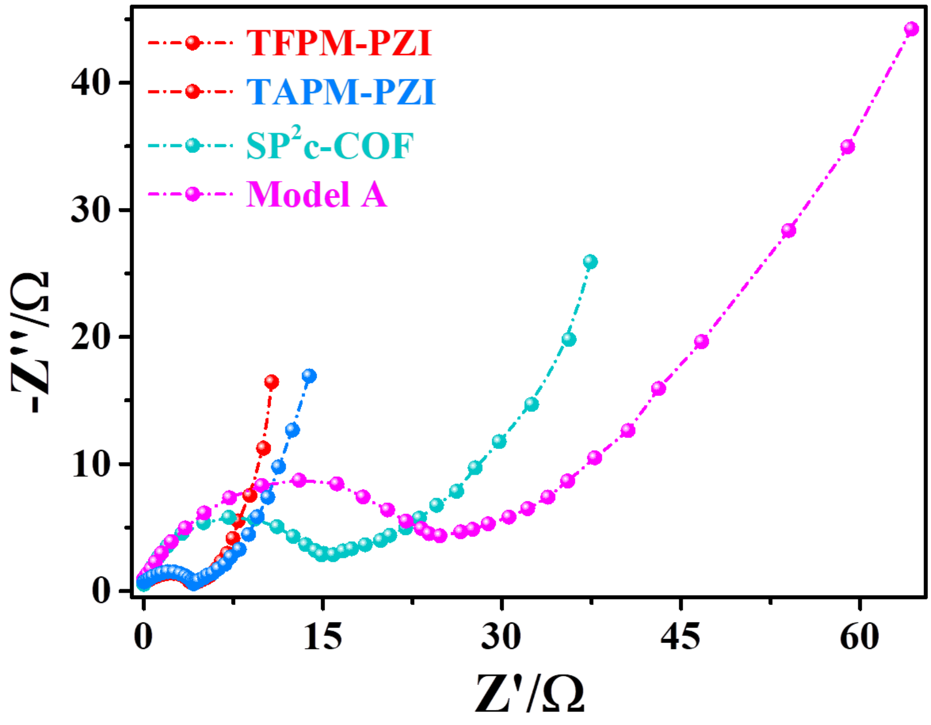

Supplementary Figure 26 | The Nyquist plots in $K_4Fe(CN)_6$ solution.

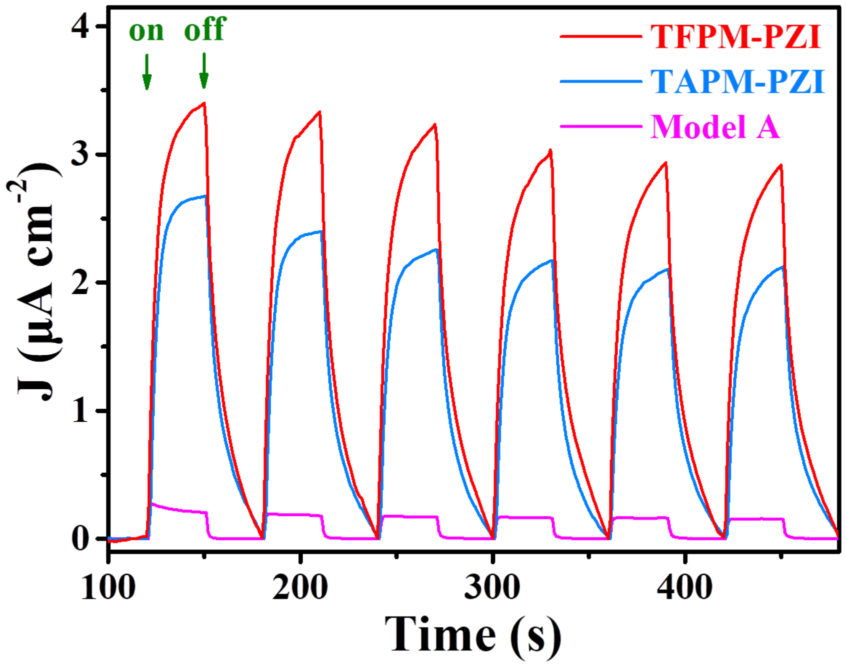

Supplementary Figure 27 | Transient photocurrent responses for TFPM-PZI, TAPM-PZI and Model A.

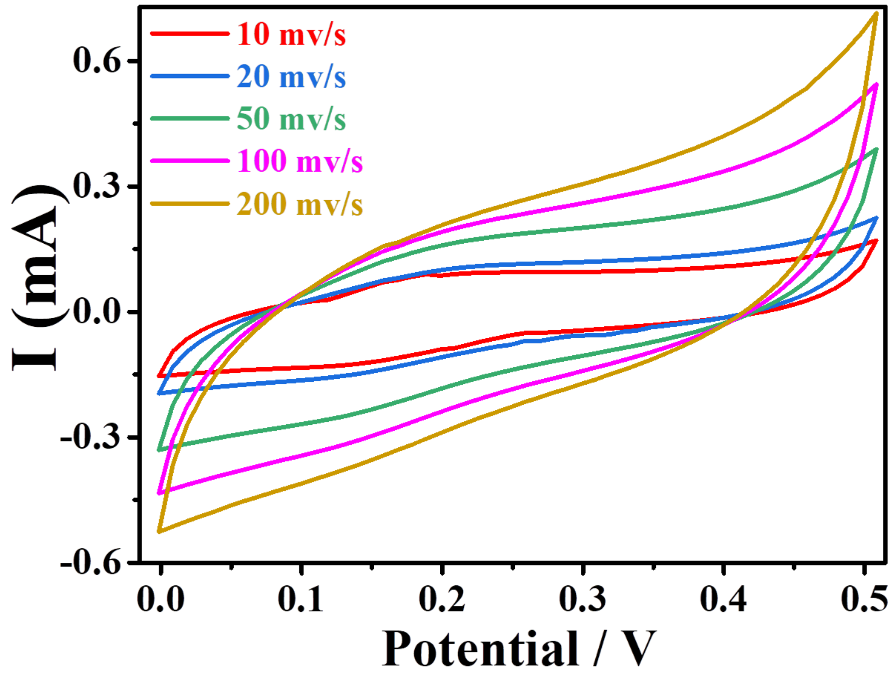

Supplementary Figure 28 | The CV curves of TAPM-PZI at different scan rate with potential ranging from 0 to 0.5V. The CV curves obtained at scanning speed and potential range within 10 ~ 200 mV/s and 0~ 0.5 V, respectively.

Clearly, no oxidation/reduction peaks were observed under the selected potential range (0 – 0.5 V), and the curves showed an approximately rectangular shape, implying the ideal electrical double layer capacitive behavior of the obtained materials[8-10]. However, the slight deviation of the ideal rectangular shape was due to the polarization effect and inherent internal resistance of the electrodes[8-10].

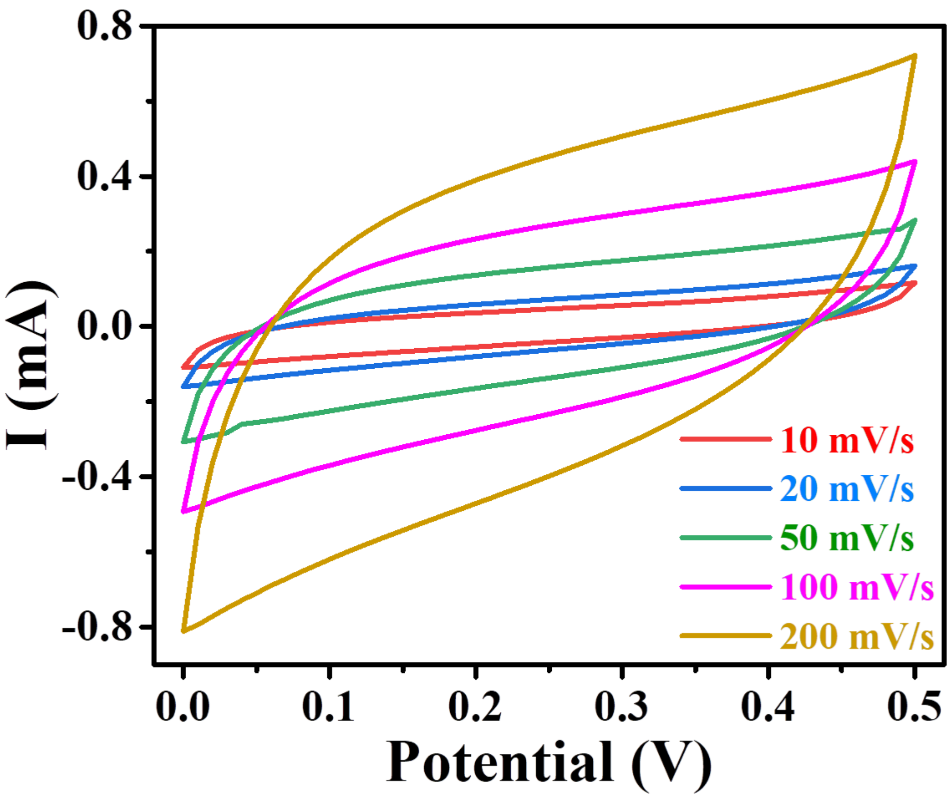

Supplementary Figure 29 | The CV curves of TFPM-PZI at different scan rate with potential ranging from 0 to 0.5V. The CV curves obtained at scanning speed and potential range within 10 ~ 200 mV/s and 0 ~ 0.5 V, respectively.

Clearly, no oxidation/reduction peaks were observed under the selected potential range (0 – 0.5 V), and the curves showed an approximately rectangular shape, implying the ideal electrical double layer capacitive behavior of the obtained materials[8-10]. However, the slight deviation of the ideal rectangular shape was due to the polarization effect and inherent internal resistance of the electrodes[8-10].

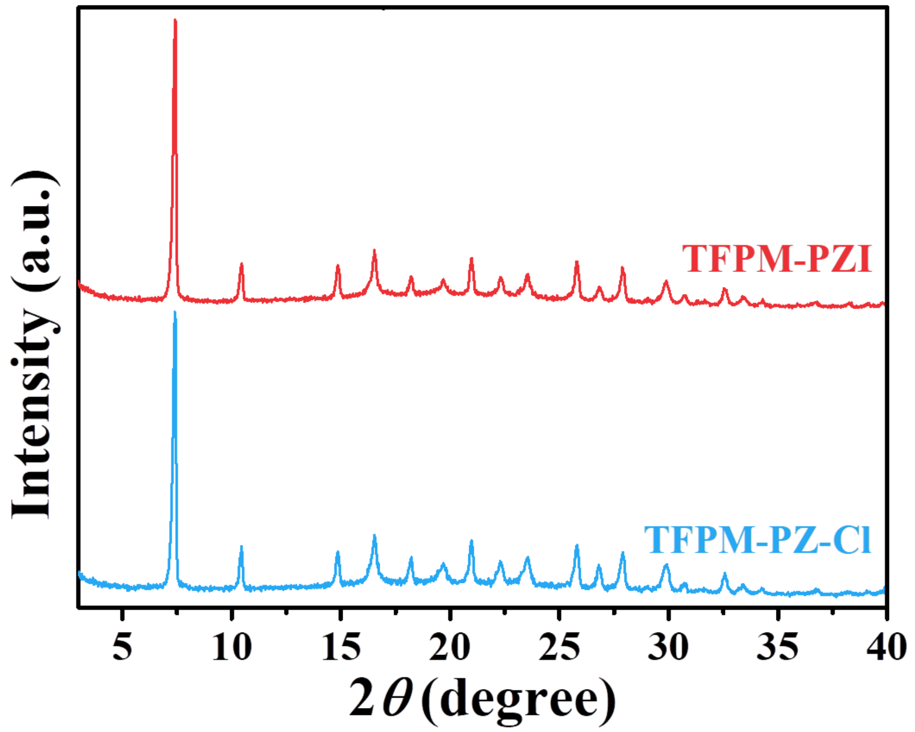

Supplementary Figure 30 | PXRD patterns of TFPM-PZI and TFPM-PZ-Cl.

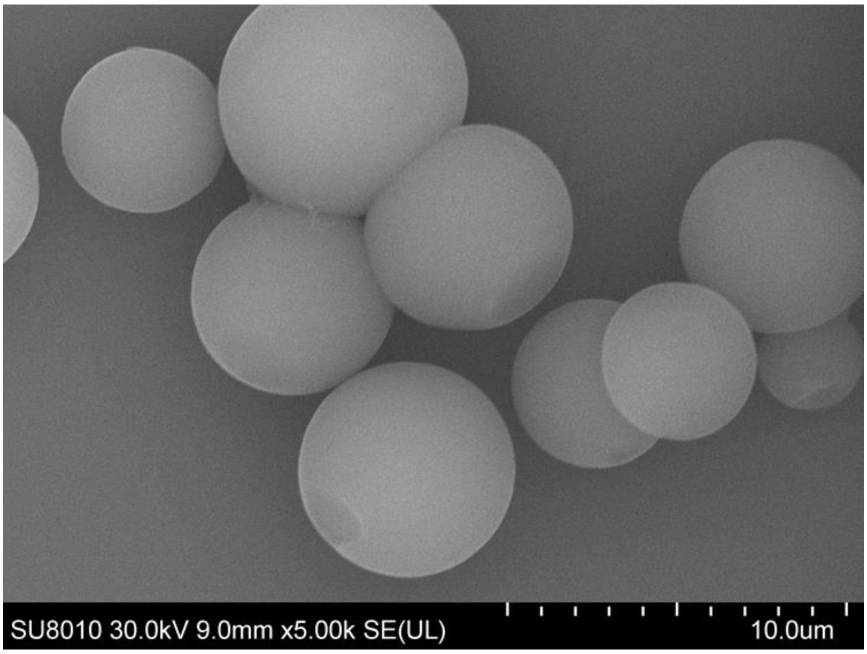

Supplementary Figure 31 | SEM image of TFPM-PZ-Cl.

.

Supplementary Table 5 | Comparison of equilibrium time and adsorption capacities of TFPM-PZ-Cl with other materials.

**Materials**	**Maximum capacity (mg/g)**	**concentration (mg/mL)**	**ReO_4^- concentration (mg/L)**	**Removal efficience (%)**	**Time**	**Refs.**
**NDTB**	49.4	0.5	48.0	**72**	36 h	11
**urea-MPN-3**	55.0	1	62.5 (0.25 mM)	**88**	30 min	12
**SCU-100**	541	1	28	**99.5**	30 min	13
**SCU-101**	217	1	28	**>95**	10 min	14
**SCU-102**	291	1	28	**>98**	30 min	15
**SCU-103**	318 ±8	1	14	**98.90**	5 min	16
**SCU-CPN-1**	999 ±20	1	28	**>99**	<30 s	17
**$DhaTG_{Cl}$**	437	1	25	**99.1**	30 min	18
**SBN**	786	0.5	28	**>99**	10 min	19
**$PQA\text{-}pN(Me)_2Py\text{-}Cl$**	1127	0.5	50	**95.3**	5 min	20
**CPN-tpm**	1133	15/250	35	**96**	20 min	21
**TFPM-PZI**	**546.1**	**0.5**	**28**	**>95**	**2 min**	**This work**

Supplementary Table 6 | Fitting results based on the Pseudo-first-order kinetic and Pseudo-second-order kinetic models.

Isotherm model	Parameters
Langmuir	q_m = 546.1 mg g^{-1}<br>K_L = 0.45 L mg^{-1}<br>R^2 = 0.988
Freundlich	K_F = 247 mg^{1-n} L^n g^{-1}<br>n = 4.16<br>R^2 = 0.95

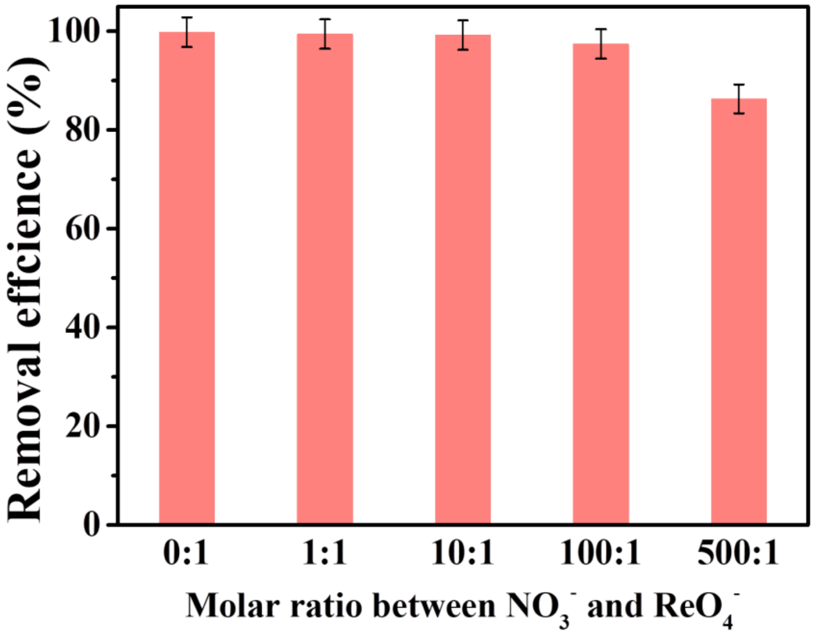

Supplementary Figure 32 | Removal effcience of ReO_4^- by TFPM-PZ-Cl in the presence of competitive anions. Error bars represent S.D. n=3 independent experiments.

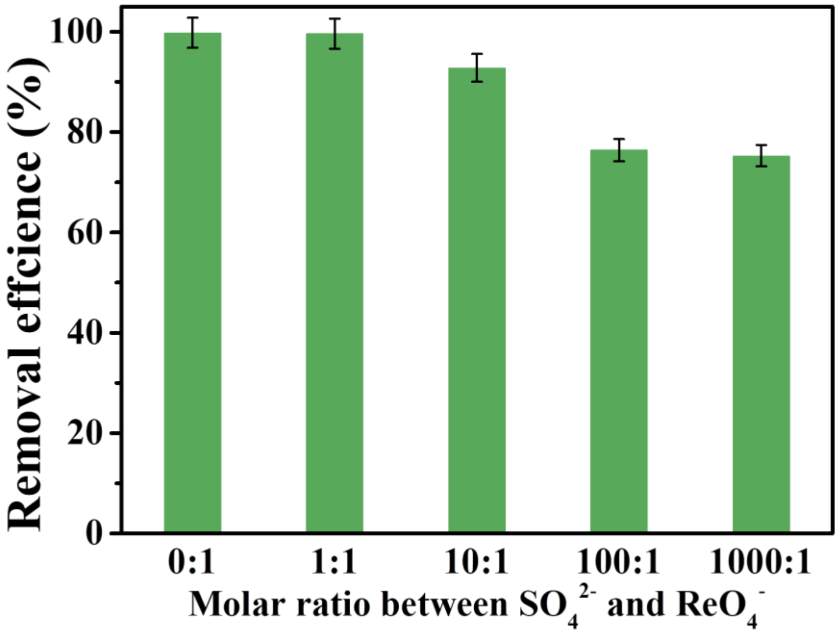

Supplementary Figure 33 | Effect of excess competing NO_3^- anions on ReO_4^- by TFPM-PZ-Cl. Error bars represent S.D. n=3 independent experiments.

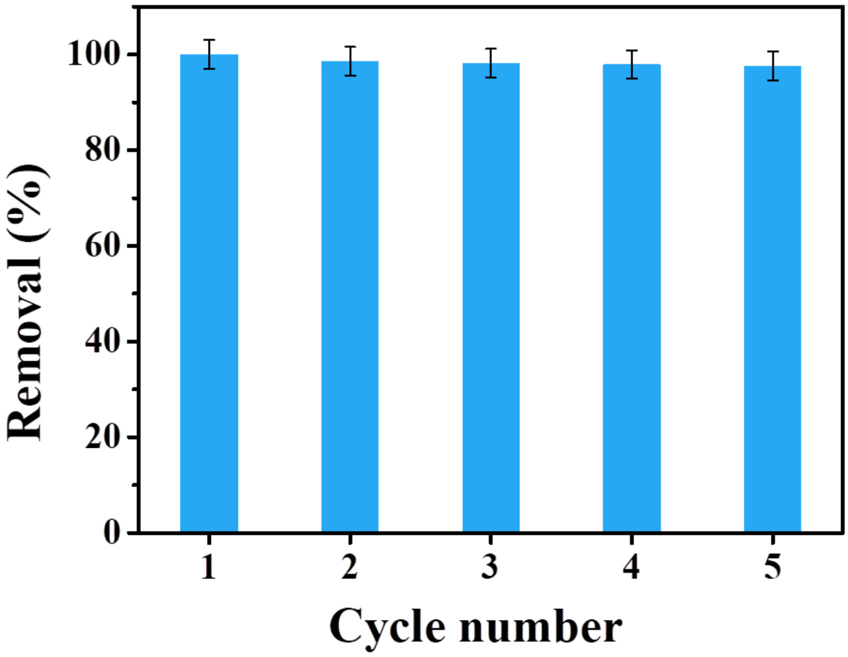

Supplementary Figure 34 | Reversibility of TFPM-PZ-Cl for removing ReO_4^- under the condition of 3 M NaCl at 80 ℃. Error bars represent S.D. n=3 independent experiments.

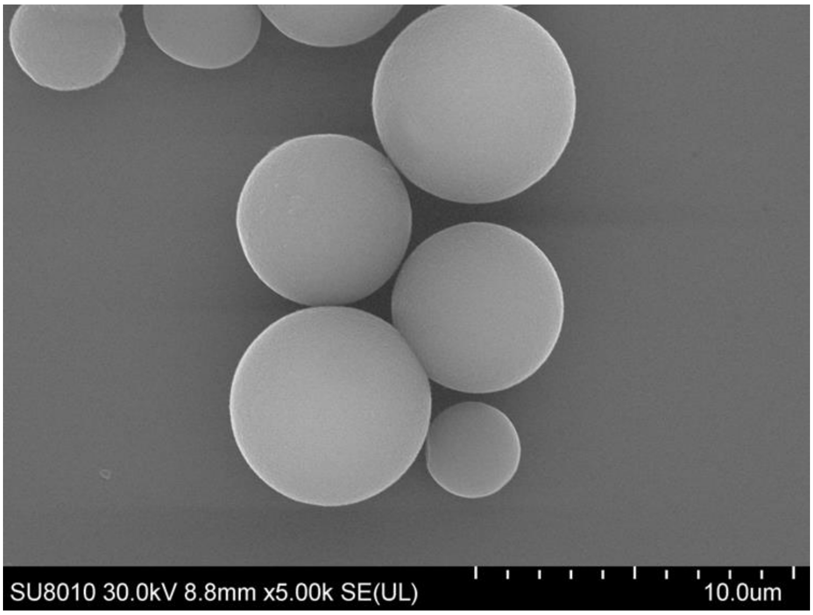

Supplementary Figure 35 | SEM image of TFPM-PZ-Cl after six cycles.

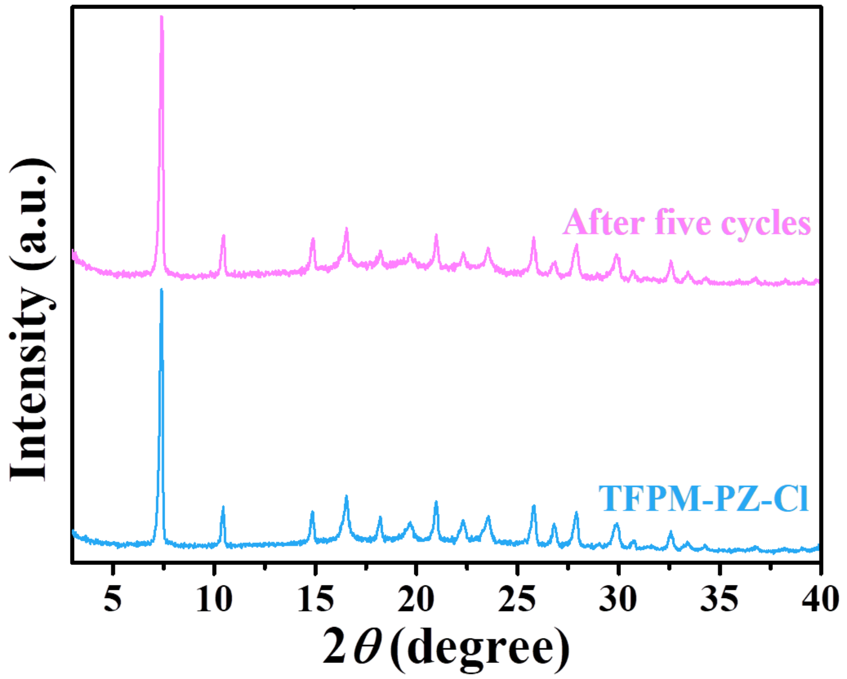

Supplementary Figure 36 | PXRD pattern of TFPM-PZ-Cl and after five cycles.

Supplementary Table 7 | Composition of Hanford LAW melter off-gas scrubber solution.

Anion	Concentration, mol/L	Anion: TcO_4^- molar ratio
TcO_4^-	1.94×10^{-4}	1.0
NO_3^-	6.07×10^{-2}	314
Cl^-	6.39×10^{-2}	330
NO_2^-	1.69×10^{-1}	837
SO_4^{2-}	6.64×10^{-6}	0.0343
CO_3^{2-}	4.30×10^{-5}	0.222

Supplementary Table 8 | Results of ReO_4^- sorption by TFPM-PZ-Cl from simulated Hanford waste.

Simulated wastes	Anions	Solid-to-liquid ratio (g/L)	Anion removal percentange (%)
Hanford waste	ReO_4^-	1：1	52.3
	ReO_4^-	5：1	86.4

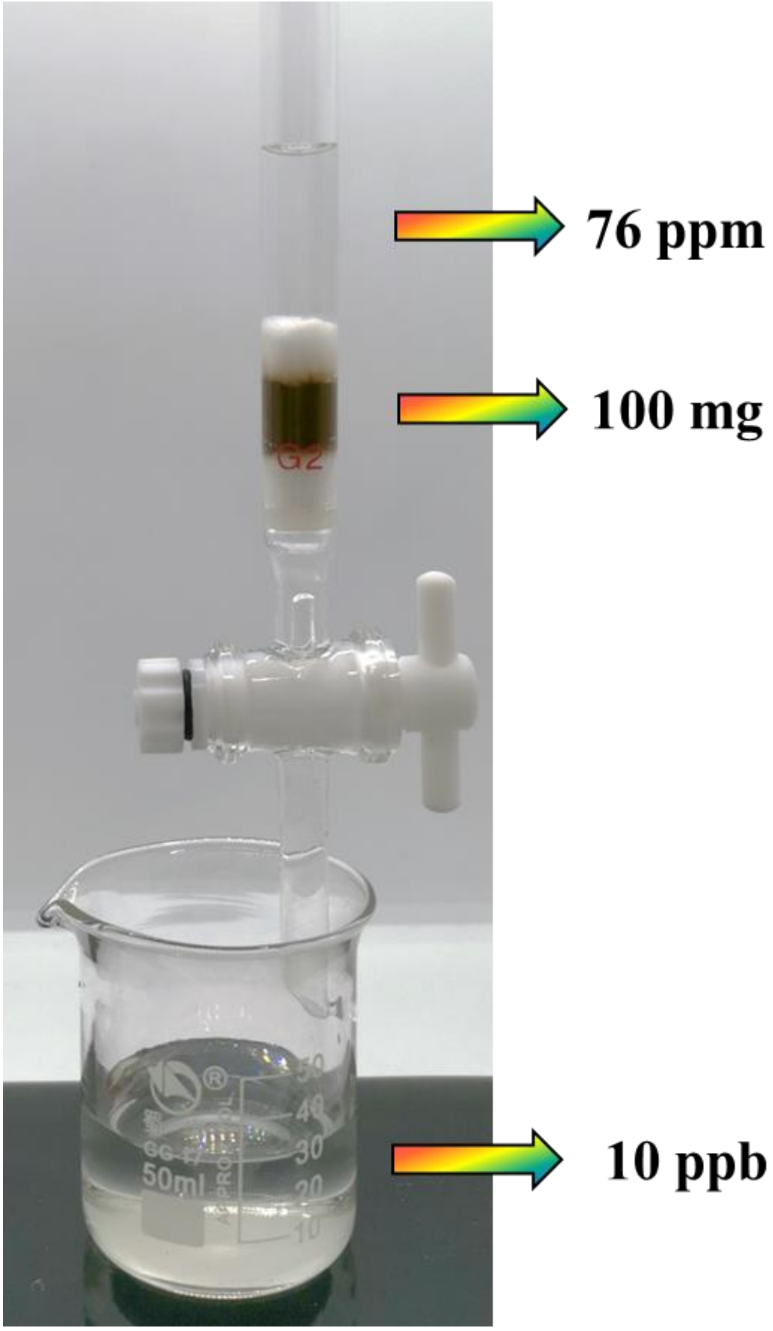

Supplementary Figure 37 | Column-chromatographic experiment for ReO_4^-. To evaluate TFPM-PZ-Cl at real application for ReO_4^- removal, a chromatographic column experiment was conducted with 100 mg TFPM-PZ-Cl as the stationary phase, and 76 ppm ReO_4^- solution as mobile phase under ambient pressure. Impressively, the ReO_4^- removal rate was high up to 99.99%, and the ReO_4^- concentration in the column eluted water was about 10 ppb.

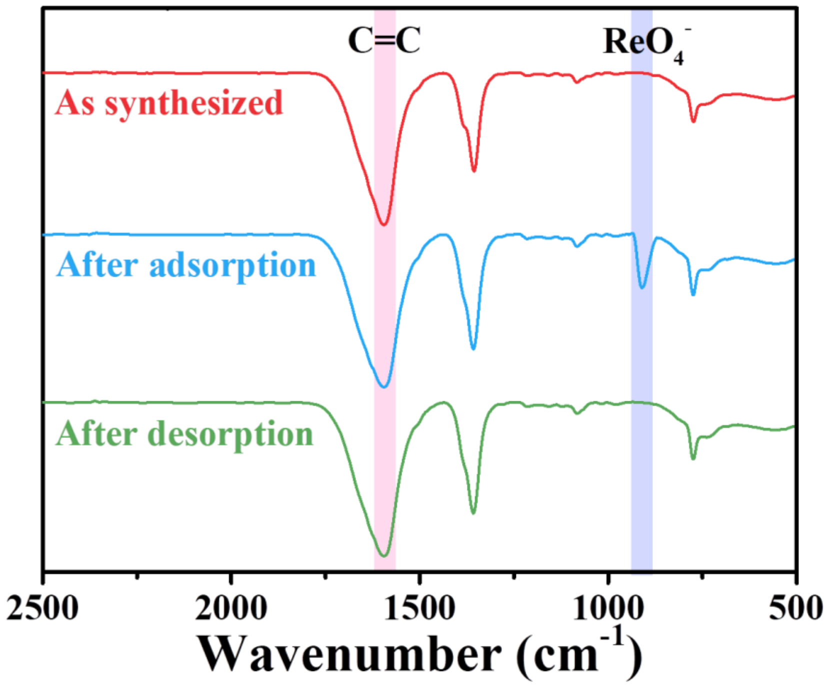

Supplementary Figure 38 | FT-IR spectra of TFPM-PZ-Cl after adsorption ReO_4^- and desorption of ReO_4^-.

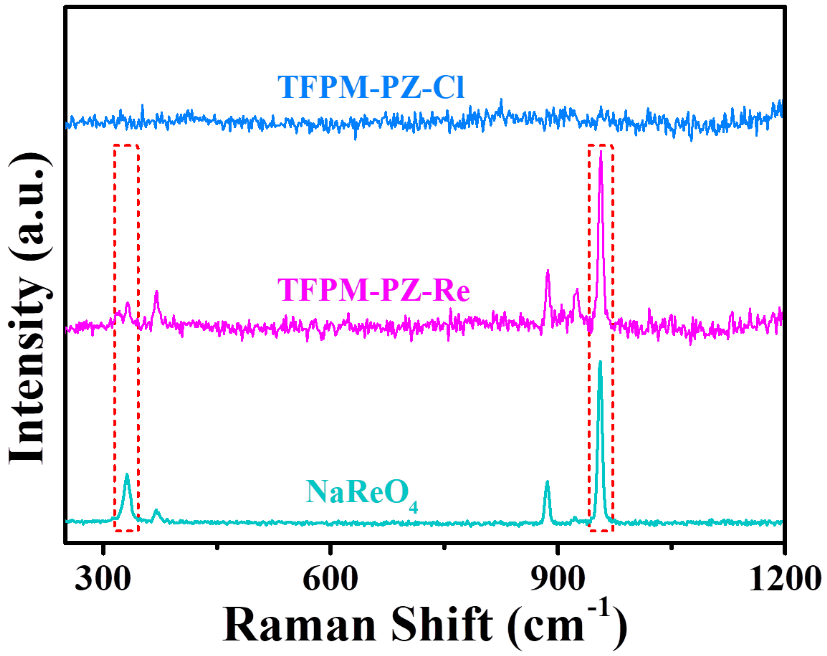

Supplementary Figure 39 | Raman spectra of the TFPM-PZI-Cl, TFPM-PZ-Re and $NaReO_4$.

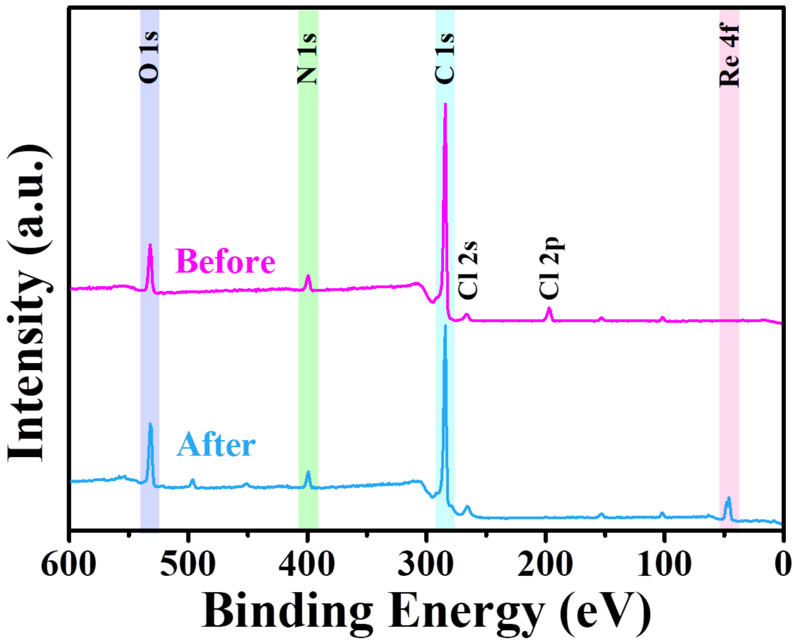

Supplementary Figure 40 | XPS survey spectra of TFPM-PZ-Cl before and after adsorbed ReO_4^-.

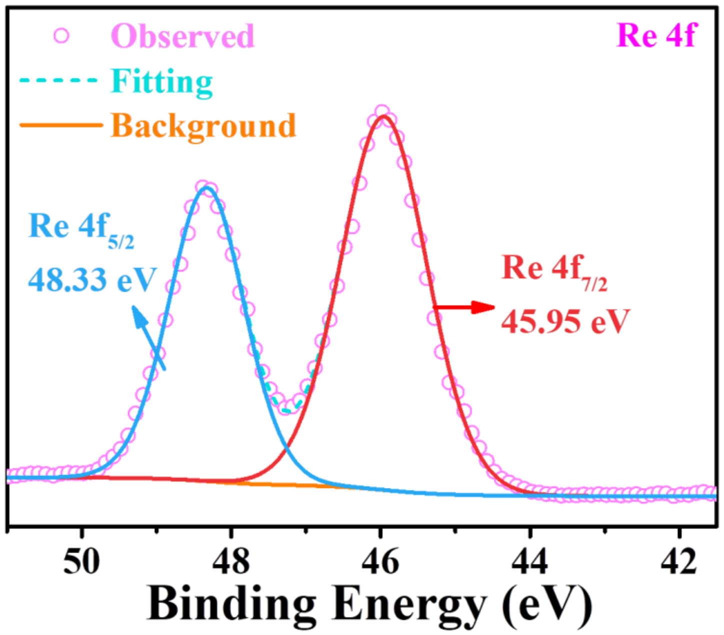

Supplementary Figure 41 | XPS analysis of Re 4f from TFPM-PZ-Re. The Re 4f core-level spectrum in TFPM-PZ-Cl was consistent with that of ReO_4^-, indicating that the Re species retains the oxidation state of Re (VII).

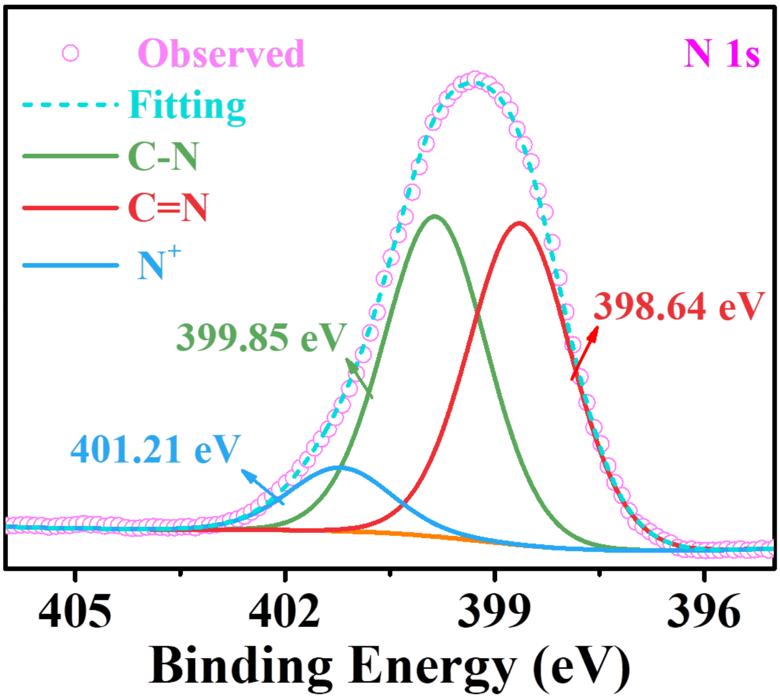

Supplementary Figure 42 | XPS analysis of N1s from TFPM-PZ-Cl.

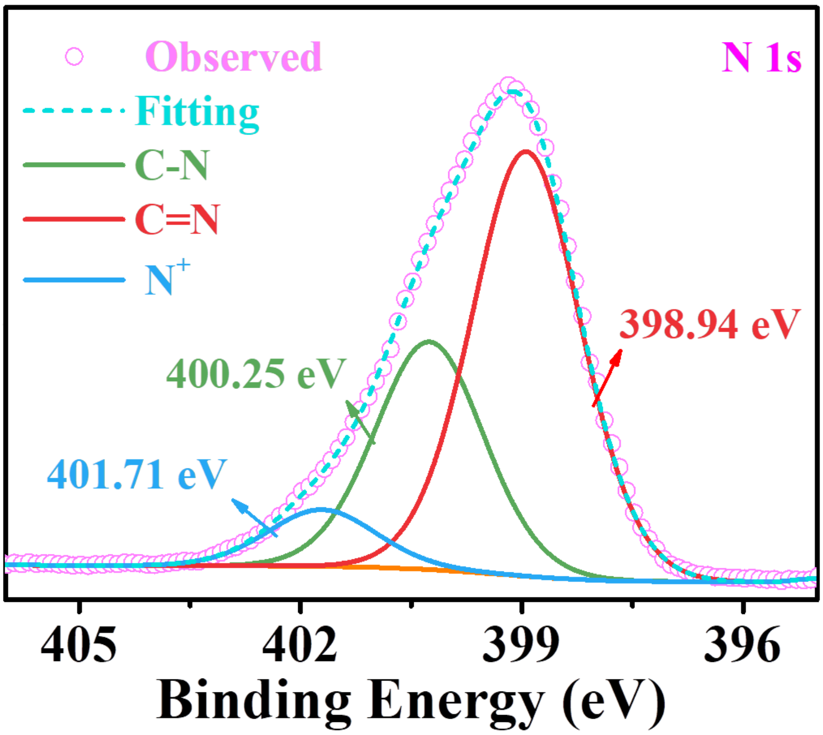

Supplementary Figure 43 | XPS analysis of N1s from TFPM-PZ-Re.

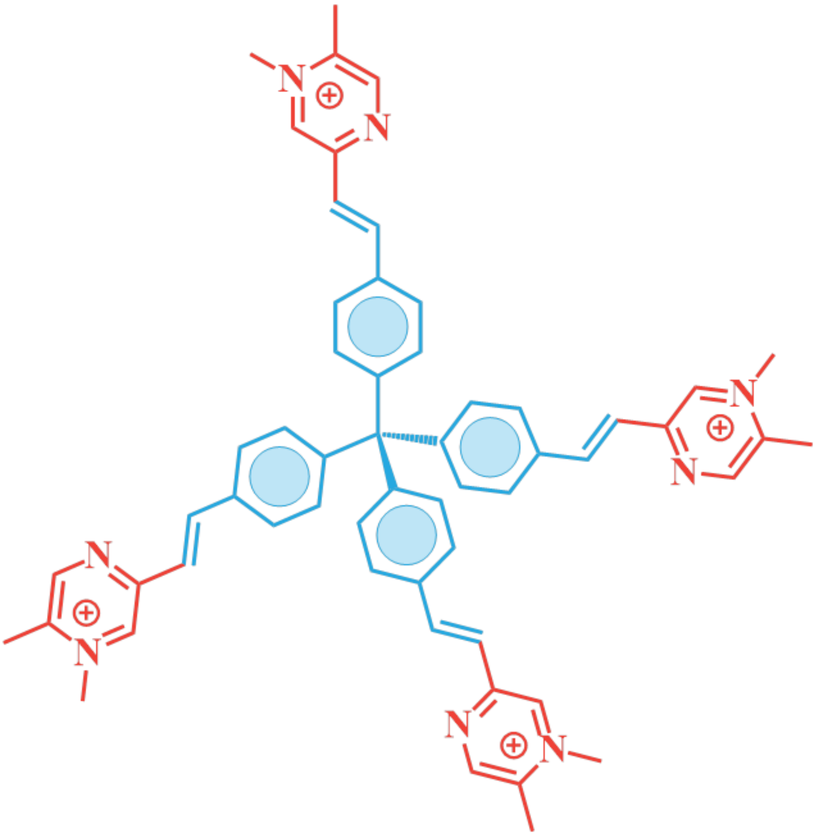

Supplementary Figure 44 | The model for describing the local structure of TFPM-PZI.

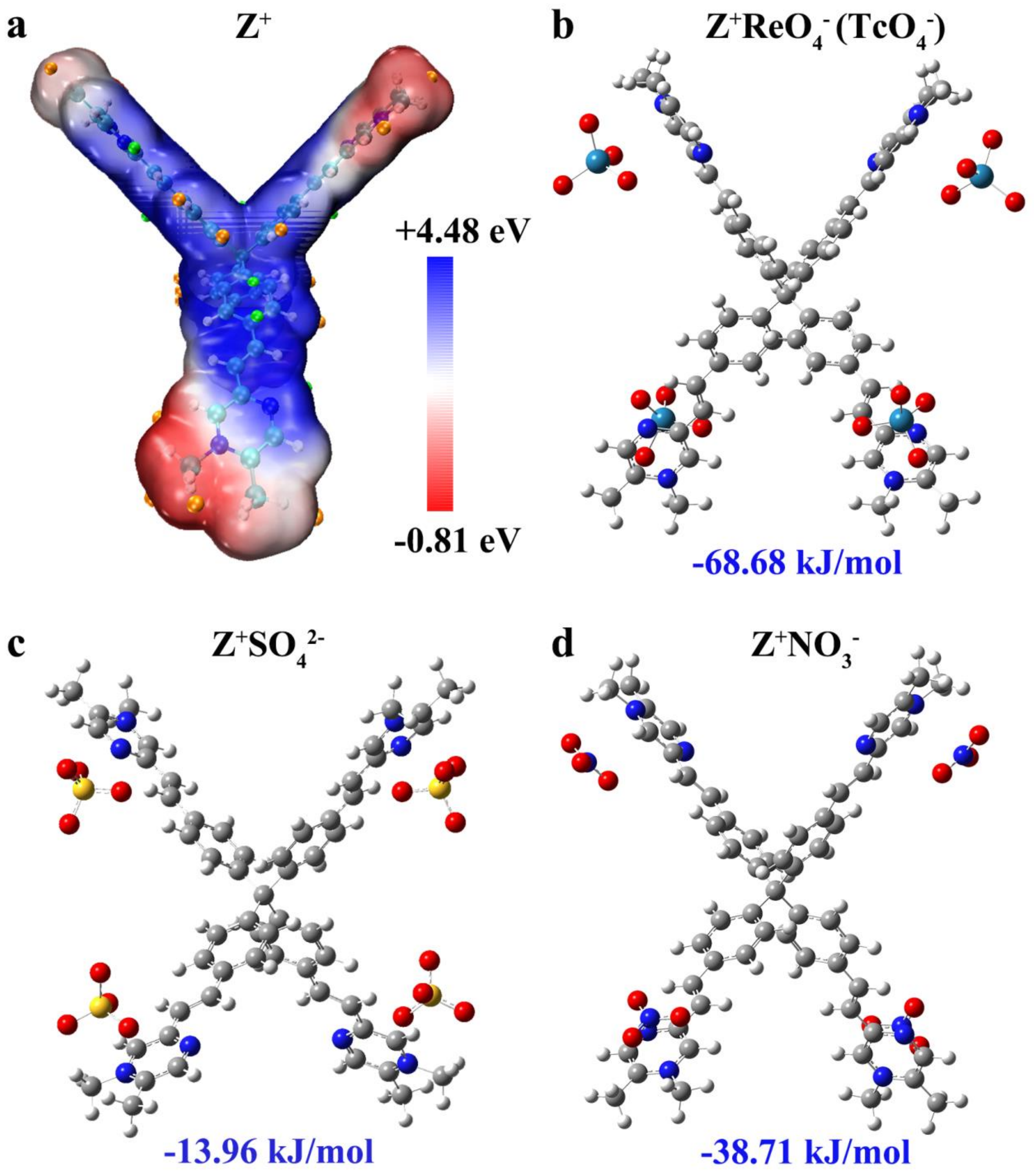

Supplementary Figure 45 | a Corresponding electrostatic potential distribution of Z^+; b Optimized structures of the Z^+ binding with ReO_4^-; c SO_4^{2-}, d NO_3^- and the corresponding binding energies.

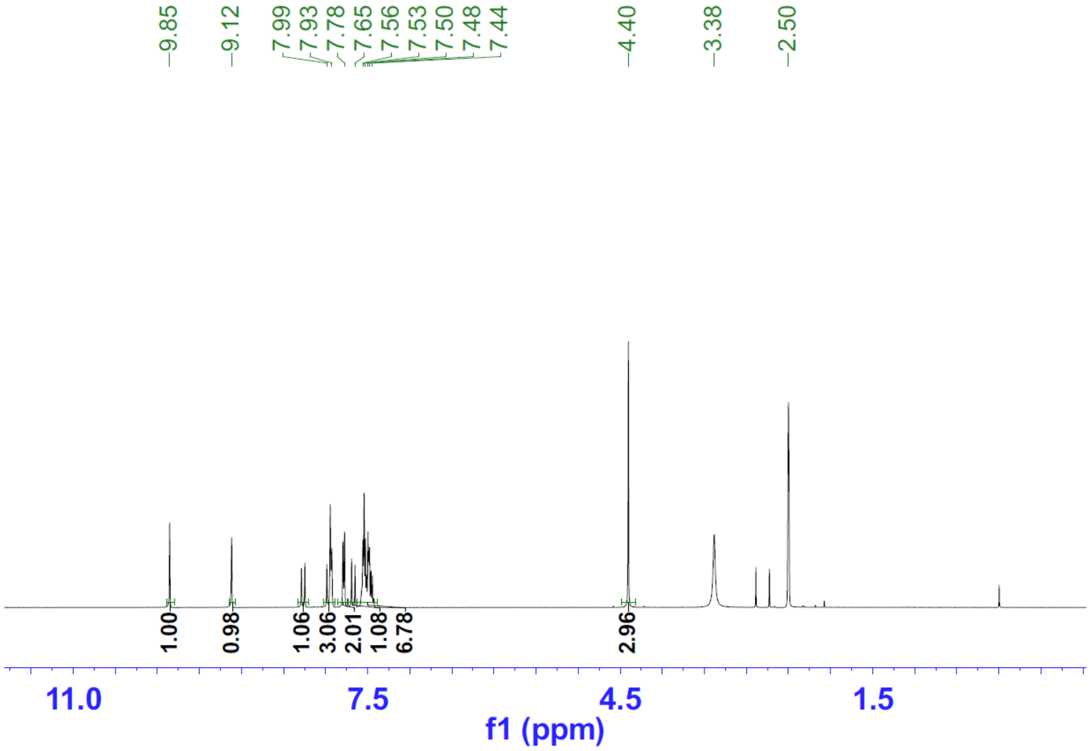

Supplementary Figure 46 | ^{1}H NMR spectra of Model compound A in DMSO-d^6.

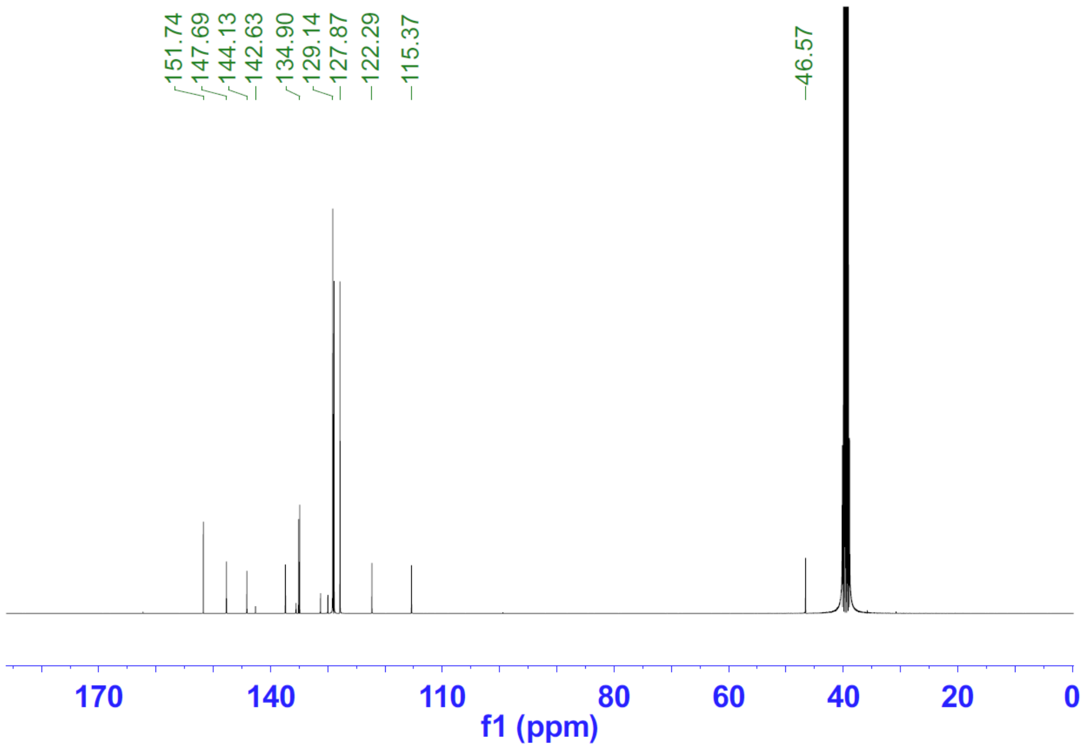

Supplementary Figure 47 | ^{13}C NMR spectra of Model compound A in DMSO-d^6.

Supporting References

1. Hess, B., Kutzner, C., van der Spoel, D. & Lindahl, E. GROMACS 4: Algorithms for Highly Efficient, Load-Balanced, and Scalable Molecular Simulation. *J Chem Theory Comp.* **4**, 435–447 (2008).
2. Jorgensen, WL. & Tirado-Rives, J. Potential energy functions for atomic-level simulations of water and organic and biomolecular systems. *P Natl Acad Sci.* **102**, 6665-6670 (2005).
3. Dodda, LS., Vilseck, JZ., Tirado-Rives, J. & Jorgensen, WL. 1.14*CM1A-LBCC: Localized Bond-Charge Corrected CM1A Charges for Condensed-Phase Simulations. *J Phys Chem B.* **121**, 3864-3870 (2017).
4. Dodda, LS., Cabeza de Vaca, I., Tirado-Rives, J. & Jorgensen, WL. LigParGen web server: an automatic OPLS-AA parameter generator for organic ligands. *Nucleic Acids Res.* **45**, W331-W336 (2017).
5. Rappe, AK., Casewit, CJ., Colwell, KS., WAG, WA. & Skiff WM. UFF, a full periodic table force field for molecular mechanics and molecular dynamics simulations. *J Am Chem Soc.* **114**, 10024–10035 (1992).
6. Essmann, U. et al. A smooth particle mesh Ewald method. *J Chem Phys.* **103**, 8577-8593 (1995).
7. Hess, B., Bekker, H., Berendsen, HJC. & Fraaije, JGEM. LINCS: A linear constraint solver for molecular simulations. *J Comput Chem.* **18**, 1463-1472 (1997).
8. Pan, M. et al. Carbon cloth as an important electrode support for the high selective electrosorption of uranium from acidic uranium mine wastewater. *Sep Purif Technol.* **281**, 119843 (2022).
9. Liao, Y., Yan, C., Zeng, K., Liao, C. & Wang, M. Asymmetric polysaccharide-bound graphene electrode configuration with enhanced electrosorption performance for uranium (VI) ions. *Chem. Eng. J.* **424**, 130351 (2021).
10. Xue, Y. et al. Electroadsorption of uranium on amidoxime modified graphite felt. *Sep Purif Technol.* **255**, 117753 (2021).
11. Wang, S. et al. NDTB-1: a supertetrahedral cationic framework that removes TcO_4^- from solution. *Angew. Chem. Int. Edit.* **49**, 1057-1060 (2010).
12. Shen, J., Chai, W., Wang, K. & Zhang, F. Efficient removal of anionic radioactive pollutant from water using ordered urea-functionalized mesoporous polymeric nanoparticle. *ACS Appl. Mater. Interfaces.* **9**, 22440-22448 (2017).
13. Sheng, D. et al. Efficient and selective uptake of TcO_4^- by a cationic metal–organic framework material with open Ag^+ sites. *Environ. Sci. Technol.* **51**, 3471-3479 (2017).
14. Zhu, L. et al. Identifying the recognition site for selective trapping of $^{99}TcO_4^-$ in a hydrolytically stable and radiation resistant cationic metal–organic framework. *J. Am. Chem. Soc.* **139**, 14873-14876 (2017).

15. Ma, Y. et al. Three-dimensional chemically stable covalent organic frameworks through hydrophobic engineering. *Angew. Chem. Int. Edit.* **59**, 19633-19638 (2020).
16. Shen, N. et al. $^{99}TcO_4^-$ removal from legacy defense nuclear waste by an alkaline-stable 2D cationic metal organic framework. *Nat. Commun.* **11**, 5571 (2020).
17. Li, J. et al. $^{99}TcO_4^-$ remediation by a cationic polymeric network. *Nat. Commun.* **9**, 3007 (2018). **2018**, *9*, 3007.
18. Da, H.-J., Yang, C.-X. & Yan, X.-P. Cationic covalent organic nanosheets for rapid and selective capture of perrhenate: an analogue of radioactive pertechnetate from aqueous solution. *Environ. Sci. Technol.* **53**, 5212-5220 (2019).
19. Zhu, L. et al. Exceptional perrhenate/pertechnetate uptake and subsequent immobilization by a low-dimensional cationic coordination colymer: overcoming the hofmeister bias selectivity. *Environ. Sci. Tech. Let.* **4**, 316-322 (2017).
20. Wang, Y. et al. Radiation controllable synthesis of robust covalent organic framework conjugates for efficient dynamic column extraction of $^{99}TcO_4^-$. *Chem* **6**, 2796-2809 (2020).
21. Li, X. et al. *3D* cationic polymeric network nanotrap for efficient collection of perrhenate anion from wastewater, *Small* ***17***, 2007994 (2021).
